# Macroscopic Electromagnetic Cooperative Network-Enhanced MXene/Ni Chains Aerogel-Based Microwave Absorber with Ultra-Low Matching Thickness

**DOI:** 10.1007/s40820-022-00869-7

**Published:** 2022-07-05

**Authors:** Fei Pan, Yanping Rao, Dan Batalu, Lei Cai, Yanyan Dong, Xiaojie Zhu, Yuyang Shi, Zhong Shi, Yaowen Liu, Wei Lu

**Affiliations:** 1 Shanghai Key Lab. of D&A for Metal-Functional Materials, School of Materials Science & Engineering, Tongji University, Shanghai, 201804 People’s Republic of China; 2 Shanghai Key Laboratory of Special Artificial Microstructure Materials and Technology, School of Physics Science and Engineering, Tongji University, Shanghai, 201804 People’s Republic of China; 3 Materials Science and Engineering Faculty, Politehnica University of Bucharest, 060042 Bucharest, Romania

**Keywords:** Electromagnetic wave absorption, Ti_3_CNT_x_ MXene, Highly oriented Ni chains, Electromagnetic cooperation, Magnetic coupling

## Abstract

Electromagnetic cooperative strategy has been presented as a mainstream approach that can effectively optimize the matching thickness of dielectric loss dominant system. However, it is still challenging for dielectric–magnetic loss coexisting-type absorber to develop electromagnetic wave (EMW) performance with ultra-low matching thickness (≤ 1 mm). Breaking the limitation of traditional electromagnetic response at microscopic/mesoscopic scale, a ficus microcarpa-like magnetic aerogel with macroscopical electromagnetic cooperative effect was fabricated through highly oriented self-assembly engineering. The highly oriented Ni chains with unique macroscopic morphology (~ 1 cm in length) were achieved via a special magnetic field-induced growth. Strong magnetic coupling was observed in the Ni chains confirmed by the micromagnetic simulation. The deductive calculation validates that maintaining high value of electromagnetic parameters at high frequencies is the prerequisites of ultrathin absorber. The electromagnetic cooperative networks with uninterrupted and dual pathways spread through the entire aerogel skeleton, resulting in the impressive permittivity even at high frequencies. Consequently, the aerogel exhibits a remarkable EMW performance at an ultrathin thickness of 1 mm. Thus, based on the modulation of electromagnetic parameters, this work proposed a macroscopic ordered structure with the electromagnetic cooperative effect useful to develop a suitable strategy for achieving ultrathin EMW absorbers.

**Supplementary Information:**

The online version contains supplementary material available at 10.1007/s40820-022-00869-7.

## Highlights

- A ficus microcarpa-like magnetic aerogel with macroscopical electromagnetic cooperative effect was fabricated through highly oriented self-assembly engineering.
- The highly oriented Ni chains with unique macroscopic morphology (~ 1 cm in length) were achieved for the first time via a special magnetic field-induced growth.
- The electromagnetic cooperative networks with uninterrupted and dual pathways spread through the entire aerogel skeleton, resulting in a remarkable electromagnetic wave performance at an ultrathin thickness of 1 mm.

**Supplementary Information:**

The online version contains supplementary material available at 10.1007/s40820-022-00869-7.

## Graphical abstract

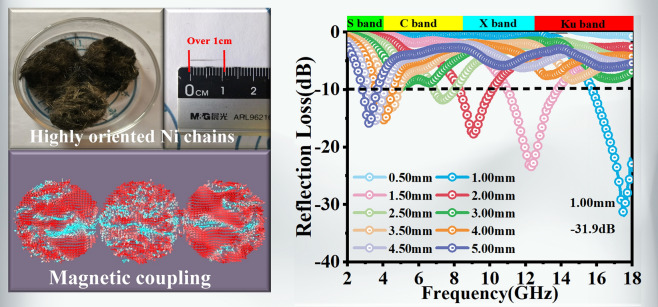

## Introduction

The increasing electromagnetic wave (EMW) interference radiation and pollution is severely threatening human health and affecting electronic instruments. In view of this situation, various traditional EMW absorbers, including graphene, carbon nanotubes, ferrite and silicon carbide, have been synthesized to address these issues [1–6]. Despite decent EMW, absorption performance has been obtained for the traditional EMW absorbers, and there are still some shortcomings, such as high filling ratio, high matching thickness and narrow effective bandwidth (EBW), which impedes their practical applications [7]. In the last few years, emerging strategies have been widely used to optimize the matching thickness of absorber, especially the idea of electromagnetic cooperation [8, 9]. EMW absorbers, coexisting dielectric loss and magnetic loss media, generate an electromagnetic cooperative loss network in the process of responding with EMW spontaneously [10]. This network not only induces magnetic loss for enriching attenuation mechanism but also produces interfacial effects due to the existence of heterogeneous interface nearby, which will affect the crystal and electronic structure [11]. After Che’s group firstly proposed this idea in 2004 [12], numerous EMW absorbers with various component collocation and structural construction were developed and explored based on the idea of electromagnetic cooperation [13, 14]. The electromagnetic cooperative effect of most absorbers mainly exists at the microscopic or mesoscopic scales. In the process of uniformly mixing with the binder, the local electromagnetic cooperation is easily interrupted by the insulating medium, thus impeding the construction of conductive electromagnetic cooperative network. However, there is no attempt to achieve uninterrupted EMW dissipative pathway in ultra-long range for further enhancing of the EMW absorption ability via electromagnetic cooperative effect at macroscale (over 1 mm). Most of the works still fail to achieve effective absorbing performance with thin matching thickness (below 1 mm).

At present, the dielectric loss and magnetic loss medium commonly used in EMW absorbing materials are mostly at micro/nanosize in morphology [15, 16]. Therefore, the integration of these micro/nanosize materials into macroscopic structures by appropriate techniques is the core of obtaining electromagnetic cooperative effect at macroscale. Self-assembly has been recognized as one of the most efficient technologies that is able to integrate materials with micro/nanosize into continuous structures with collective physicochemical properties [17]. Therefore, the driving force of freeze-drying technology and ice-templated assembly has the advantages of high efficiency, universality and high controllability for the structure of the synthesized dielectric loss-type absorber. Orientated freezing technology can prepare aerogels with porous and three-dimensional structure at macroscopic scale. Recently, the thriving family of metal carbide and metal nitride materials (MXenes) stand out as a two-dimensional (2D) dielectric loss-type absorber in the field of EMW absorption own to their unique integration of metallic characteristic, remarkable conductivity, high specific surface area and facile tunable properties [18, 19]. On the basis of self-assembly engineering and Maxwell–Garnett theory, MXenes with aerogel structure are advantageous to the conduction and attenuation of interior EMW compared with the solid counterparts [20]. On the one hand, porous structure replaces the original internal medium with air, leading to the improved impedance matching and the optimization of the dielectric constant [21]. On the other hand, self-assembly process avoids the aggregation of MXenes, thus forming a macroscopically conductive loss network in ultra-long range via van der Waals force between the lamellas [22]. To further improve the orientation of the assembled MXenes at macroscale, directional freeze drying has been investigated. The resulting structure is oriented in the direction of the temperature gradient and disordered in a plane perpendicular to the temperature gradient [23]. It is anticipated that MXenes lamellas will form an overall disordered and locally oriented arrangement structure, which further reinforce the charge migration along the macroscopic plane direction. When dispersed in the binder, directional freeze-drying technology enables MXenes to transform from discontinuous dielectric loss network at micro/nanoscale to continuous network with macroscopic ultra-long-range order along temperature gradient direction.

In addition to the construction of dielectric loss network at the macroscale, obtaining the macroscopic magnetic loss network is more difficult because the effective valence bond or binding between magnetic medium to achieve bottom-up assembly is absent [24]. Regarding the current situation, it is necessary to apply an external driving force during the preparation process to achieve self-assembly in magnetic materials. Magnetic field-induced self-assembly is considered a feasible solution to prepare highly oriented magnetic materials, which can transfer energy without contact to the microscopic scale of matter [25]. Highly oriented magnetic materials with good dispersion of fillers (chain, needle, nanowire, nanofiber, etc.) and high aspect ratio are recommended to construct three-dimensional polarization network, thereby considering as a suitable EMW absorber. The low percolation threshold of 1D structures also enables charge transfer and conduction on highly oriented magnetic materials. Besides, according to our previous studies [26], the magnetic chains formed by the directional alignment of magnetic nanoparticles exhibit strong electromagnetic coupling effects resulted from interlacing magnetic flux field effect. As the length of chain increases, the coupling interaction becomes stronger, strengthening the magnetic loss. Currently, there is yet no report in the field of EMW materials on 1D magnetic materials with macrodimension in length (up to centimeter). Usually, the intensity of the external magnetic field influences the degree of magnetization of magnetic particles, thus affecting the interaction force between magnetic dipoles; the force between magnetic dipoles should be strong enough to ensure that more magnetic particles attract each other and align to form a macroscale structure. Using of conventional magnetic field to induce a self-assembly process is difficult, as it requires to generate a strong enough magnetic field during assembly. Also, forcing new equipment has to be designed for magnetic materials to achieve the macroscopically oriented self-assembly [27].

**Fig. 1:**
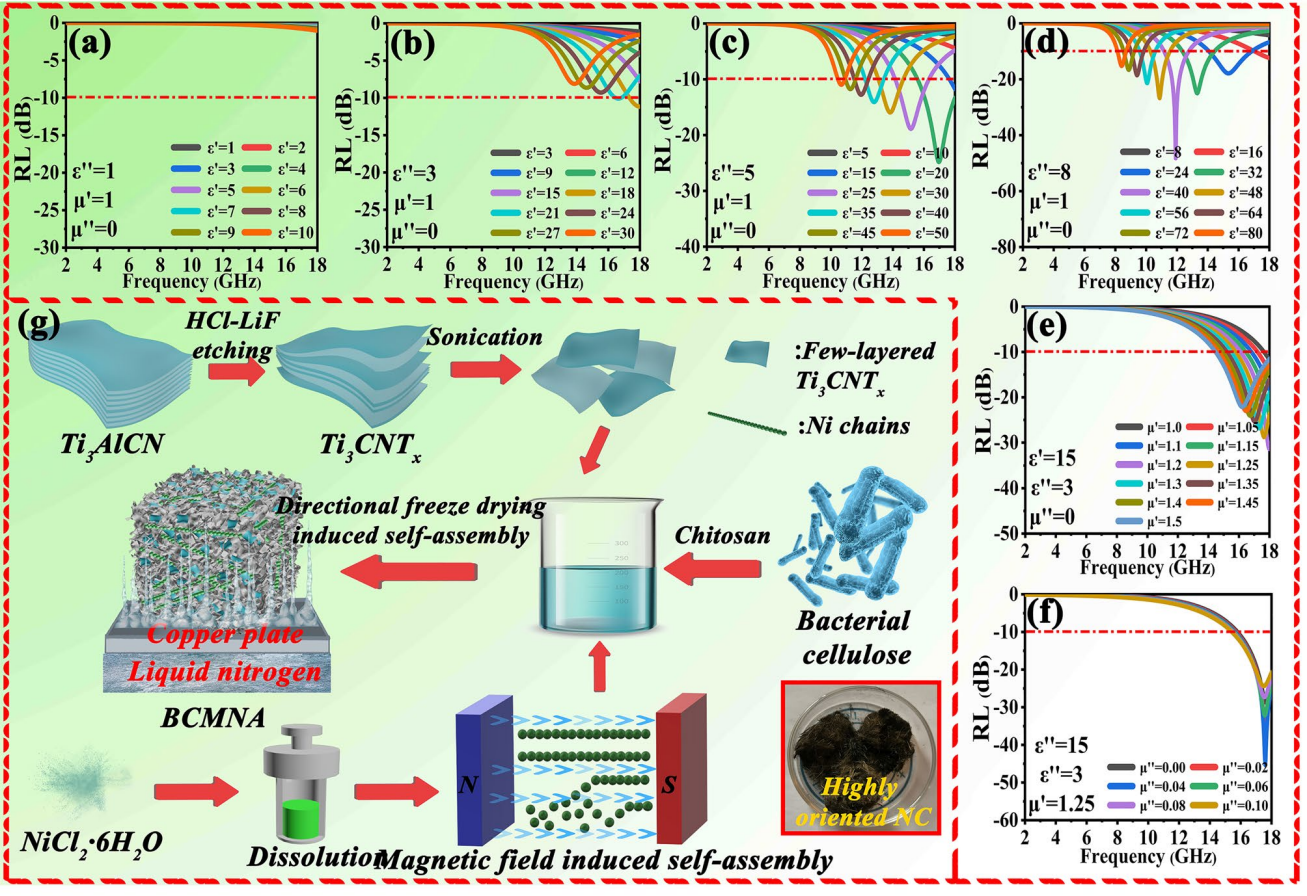
**a**–**f** Deductive calculation of reflection loss under 1 mm thickness; **g** schematic illustration for the fabrication of BCMNA

Considering the presented aspects, via macroscopically oriented self-assembly engineering, including magnetic field-assisted and directional freeze-drying-assisted self-assembly, we obtained a ficus microcarpa-like magnetic aerogel based on bacterial cellulose, Ti_3_CNT_x_ MXene and Ni chains. The highly oriented Ni chains with hairlike macroscopic morphology (~ 1 cm in length) were fabricated via a unique magnetic field-induced growth, exhibiting strong anisotropy and strong magnetic coupling as observed in the micromagnetic simulation. Tree-branch-like bacterial cellulose as the corbelled unit generates an interconnected skeleton, in which leaves (Ti_3_CNT_x_) and vines (Ni chains) are packaged inside. The 3D bacterial cellulose framework not only reinforces mechanical properties of final hybrid aerogel, but also avoids the stacking of MXene lamellae. Besides, when combined with MXenes, the dispersion of nanocellulose-chitosan will package the MXene lamellaes tightly inside. This packaging engineering optimizes the impedance mismatch of pure MXenes to some extent because EMW preferentially contact nanocellulose-chitosan framework with high impedance, thus avoiding the reflection of EMW at the interface. This packaging design also protects MXene from direct interaction with oxygen after drying, improving the durability of MXene-based aerogel. This core–shell packaging structure and assembly from 2 to 3D gives full play to the advantages of multidimensional hybrids. The unique 2D lattice structure of MXene lamellae with nanocellulose–chitosan packaging gives rise to the anisotropy of electric conductivity and polarization relaxation at heterogeneous interfaces, which will lead to reinforced EMW capability. As expected, the magnetic aerogel achieves a EMW performance with a minimum reflection loss (*RL*_min_) of − 52.5 dB at a thickness of 1.75 mm and a broad effective absorption bandwidth (EAB) of 7.0 GHz at a thickness of 2.05 mm. With the increased concentration of Ni chains, a *RL*_min_ of − 31.9 dB is achieved at the ultra-low thickness of 1 mm. The deductive calculation validates that maintaining high value of electromagnetic parameters at high frequencies is the prerequisites of ultrathin absorber (Fig. 1a–f). The excellent EMW absorption performance is attributed to the multieffect synergistic loss strategy, including progressive conductive loss network, multicomponent-induced polarization loss, porous-derived multiple scattering and especially macroscopic scale-enhanced electromagnetic cooperation. This macroscale electromagnetic cooperative network enables the absorber the ability to maintain continuous electromagnetic dissipation over centimeter-scale range, thus keeping permittivity large even at high frequency range for achieving ultrathin characteristics. This work is dedicated to the development of novel EMW materials from aspects of electromagnetic cooperative effect at macroscale, providing a brand new idea for the follow-up works.

## Experimental

### Materials

All chemical reagents were analytical grade and used as received without further purification. Specifically, chitosan (C_6_H_11_NO_4_X_2_), acetic acid glacial (C_2_H_4_O_2_), nickel(II) chloride hexahydrate (NiCl_2_·6H_2_O), hexamethylenetetramine (C_6_H_12_N_4_), sodium hydroxide (NaOH), 1,2-propanediol (C_3_H_8_O_2_), hydrochloric acid (HCl), lithium fluoride (LiF) were purchased from Sinopharm Chemical Reagent Co., Ltd., Beijing, China. Ti_3_AlCN powders (> 98 wt% purity) were purchased from Laizhou Kai Xi Ceramic Materials Co., Ltd. Bacterial cellulose dispersion was purchased from Guilin Qihong Technology.

### Preparation of Few-Layered Ti_3_CNT_x_ MXenes

A typical HCl–LiF etching method was employed to prepare Ti_3_CNT_x_ MXenes according to previous work [19]. In the first step, 0.5 g of LiF was dissolved into 10 mL of HCl solution for preparing etching solution (36.5 wt%). Then 1 g of Ti_3_AlCN was slowly added into the etching solution and hold at 50 °C for 24 h with continuously stirring. The obtained suspension was washed with deionized water though a centrifugation method (3500 rpm, 5 min) until the pH value of the supernatant became closed to 5–6 units. Finally, for obtaining few-layered MXene, the solution was ultra-sonicated for 1 h to exfoliate the MXene and preserved at low temperature.

### Preparation of Highly Oriented Ni Chains

9 mmol of NiCl_2_·6H_2_O, 16.2 mmol of hexamethylenetetramine and 2.7 mmol NaOH were added into 40 mL of 1,2-propanediol and stirred continuously for 1.5 h until completely dissolving. Next, the aqueous solution was transferred to a 50-mL Teflon-lined stainless-steel autoclave and placed into a custom magnetic heat treatment furnace, under a 0.15 T magnetic field. When cooling down to room temperature, the resultant gray flocculent was washed with deionized water and alcohol several times until the organic solvent was totally removed and then dried in vacuum at 60 °C to obtain the Ni chains.

### Preparation of Bacterial Cellulose/MXene/Ni Aerogel

Initially, 75 mg chitosan was dissolved into 10 g dilute acetic acid solution (3 mL of acetic acid + 47 mL of deionized water) and stirred for 0.5 h until solution clarification, after which suspension was mixed with 10 g of bacterial cellulose dispersion. Subsequently, 5 mL few-layered Ti_3_CNT_x_ solution and 100 mg Ni chain were added into the mixture under stirring. Then, the slurry was poured into a plastic mold for subsequent directional freeze-casting using ice template from the bottom to the top on the frozen copper plate. Then, the hydrogel was frozen in the refrigerator overnight and then freeze-dried at − 80 °C for 3 days to obtain BCMNA-1. The aerogel was named as BCMNA-2 when the process involved 200 mg Ni chains addition. BCMNA-1 and BCMNA-2 are both called BCMNA for simplifying representations. For comparison, the aerogel was named as BCA when the process involved no Ni chains and MXene addition.

### Characterization

X-ray diffraction (XRD) patterns are obtained with DX-2700 X-ray diffractometer (Cu-Ka radiation, *λ* = 1.54 Å). The morphology of the as-prepared samples was measured using scanning electron microscopy (SEM) and transmission electron microscopy (TEM). Raman spectra were recorded on a cryogenic matrix using a 532-nm laser. Fourier transform infrared (FT-IR) spectra were measured with a Thermo Scientific Nicolet iS5 spectrometer, with a resolution of 4.000 cm^−1^ in the range of 400–4000 cm^−1^, using the attenuated total reflection mode. The room magnetic temperature hysteresis loops were obtained on a vibrating sample magnetometer (VSM, manufactured by Lakeshore, Inc.). The chemical states and surface components of the samples were measured by X-ray photoelectron spectroscopy (XPS) on an Thermo Scientific K-Alpha spectrometer. Based on coaxial-line theory, the related EMW parameters of samples in the frequency of 2–18 GHz range were measured on a vector network analyzer (VNA, 3672B-S, Ceyear). Samples were wrapped into paraffin at a filling ratio of 15 wt% and then shaped into a toroid with 7 mm outer diameter and 3.04 mm inner diameter. The infrared thermal images (FLIR ONE PRO) were taken to visualize the heat insulation process of the samples on a heating platform. Micromagnetic simulation and cross-section (RCS) simulation were described in detail in the supporting information.

## Results and Discussion

### Formation Mechanism of BCMNA

**Fig. 2:**
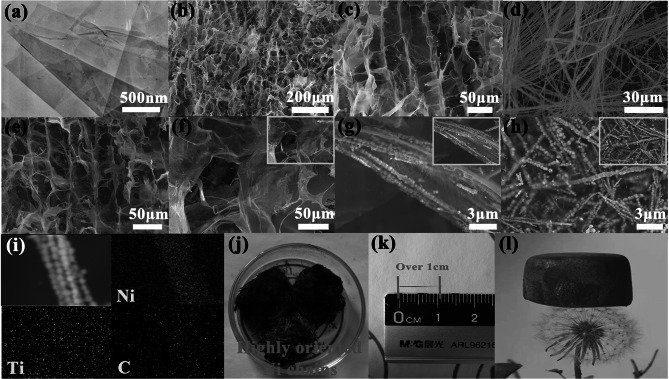
TEM and SEM images of **a** few-layered Ti_3_CNT_x_, **b** BCA, **c** BCMA, **d** highly oriented Ni chains, and **e**–**h** BCMNA-1, and blue, orange and white colors represent Ti_3_CNT_x_, Ni chains and bacterial cellulose, respectively; **i** EDS maps of BCMNA, **j**, **k** digital images showing the highly oriented Ni chains, **l** digital image showing the BCMNA-1 large sample standing on a dandelion

The ficus microcarpa-like BCMNA was prepared via the in situ magnetic field-assisted growth and following directional freeze-drying process, as exhibited in Fig. 1g. In the initial step, multiple layered Ti_3_CNT_x_ with an accordion-like structure was synthesized by HF acid etching process using Ti_3_AlCN as raw material, and the correlative microscopic crystal structure transformation is shown in Fig. S1. After subsequent ultrasonic exfoliating treatment, 2D few-layered Ti_3_CNT_X_ lamellas were obtained; their thickness can be controlled by tailoring the power and time of ultrasound. Then, under the action of magnetic field (Fig. S2a), the highly oriented Ni chains are formed by the reduction reaction of Ni^2+^ with hexamethylenetetramine and NaOH under 1,2-propanediol solvent. During this process, the OH^−^ is quenched when reacted with 1,2-propanediol, resulting CH_3_C·OHCH_2_OH with strong reducing ability, which will stimulate the reduction of Ni^2+^ via conventional reduction reaction [25]. The final magnetic chains have a highly oriented macroscopic morphology with hairlike macroscopic morphology (~ 1 cm in length, Fig. 2k) that is quite distinct from the analogs reported previously (Fig. S2b). This versatile strategy can also enable the fabrication of 1D magnetic materials (Fe, Co, ferrite, etc.), which can be used to explore the effect of magnetic field on the internal structure of the material, as well as the corresponding EMW performance. Finally, aerogels are prepared by mixing several substances into a plastic mold, in which bacterial cellulose–chitosan framework is used as the major building blocks and MXene and Ni chains are used as functionalized units. To explore the effect of the MXene and Ni chains on the EMW absorption performance of BCMNA, they are compared with samples denoted as BCA (without MXene and Ni chains), BCMA (without Ni chains) and NC (pure Ni chains).

### Microstructure and Composition of BCMNA

To observe the detailed morphology and microstructure of the samples, TEM and SEM images are analyzed (Figs. 2 and S3). The as-synthesized Ti_3_CNT_X_ displays a transparent wrinkled membrane-like structure (Fig. 2a), indicating the successful exfoliation of multilayered Ti_3_CNT_X_ into few-layered Ti_3_CNT_X._ Figure 2b exhibits the interlinked three-dimensional porous structure of the BCA due to the sublimation of ice crystals during the directional freeze-drying process. It can be noticed that the BCA is uniformly interwoven by layered channels arranged along parallel ice templates and layered walls with a cross-linked structure perpendicular to the direction of the channels. In the nanocellulose/chitosan framework, numerous thin sheets of bacterial cellulose act as the supporting skeleton, and they are uniformly interlinked with chitosan which function as the filling matrix. After the introduction of MXene, the dark lamellas are clearly visible in Fig. 2c, when compared with Fig. 2b. The bacterial cellulose suspension is in full contact with MXene before freeze-drying process, thereby hindering the agglomeration between lamellas, and leading to the formation of core–shell structure of the prepared aerogel, where the white bacterial cellulose film integrally wraps the dark MXene lamellas. Figure 2d shows the highly oriented Ni chains, with the length over 200 μm while the typical size of each nanoparticle unit is about 500 nm, which forms a novel highly oriented macroscopic morphology (Fig. 2j). When the external magnetic field is applied, the nanoparticles self-assemble to form Ni chains, which facilitates the propagation and dissipation of EMW energy due to the radial anisotropy and magnetic coupling of 1D materials. Figure 2e–h is SEM images of BCMNA-1 at different magnifications, where the blue (few-layered Ti_3_CNT_X_) and white (presents bacterial cellulose) colors are employed to easily distinguish the different components; the images show that Ni chains can uniformly intermix with the bacterial cellulose/MXene skeleton and form a ficus microcarpa-like structure. Briefly, bacterial cellulose, MXene lamellas and Ni chains act as branches, leaves and vines in this structure, respectively. The bacterial cellulose framework with good mechanical property served as the principal part of the aerogel where MXene lamellas are tightly anchored in interior, as well as uniformly decorated with Ni chains. The whole morphology is similar to a ficus macrocarpa structure, where numerous leaves are fixed to the branch, accompanying with vertically distributed vines. The high-magnification SEM image further exhibits that Ni chains in aerogel have two distributional states, a parallel distribution and a staggered one. Closed connection between Ni particles and various interaction between Ni chains changes the motion state of the internal magnetic moment and the distribution of the surrounding magnetic induction lines, generating diverse electromagnetic coupling networks inside the aerogel and promoting the attenuation of EMW. The related energy-dispersive X-ray spectroscopy (EDX) mapping also confirms that Ni, C and Ti elements are present in the BCMNA-1 (Fig. 2i). The digital images of highly oriented NC are displayed in Fig. 2j–k, where hairlike macroscopic morphology can be clearly observed and each chain has a length over ~ 1 cm, longer than any current magnetic chains. Thanks to the large presence of the air in the aerogel, BCMNA-1 has remarkable ultra-low density (12.94 mg cm^−3^), which enables new potential applications in civil and military sectors. As shown in Fig. 2l, the BCMNA-1 can lie on top of a dandelion without breaking the fluffy seed heads. Moreover, BCMNA-1 also exhibits perfect load-bearing capacity due to the synergy between bacterial cellulose and chitosan. When gradually increasing the mass of the weight placed on the top of BCMNA-1 (Fig. S4), it is seen that the aerogel can hold up to 10–200 g of weight without deformation, which is 50–1,000 times of its own mass (200 mg). The hydroxyl group of bacterial cellulose and the amine group of chitosan can form an effective hydrogen bond to enhance the binding of the matrix. Besides, chitosan can promote dispersion between cellulose and improve the internal structural uniformity of aerogel, thus making it soft, durable and load bearing. It can be deduced from the aerogel without the addition of chitosan or bacterial cellulose that the bacterial cellulose determines the ability of aerogel formation, while the chitosan compacts the connections between cellulose sheets, further optimizing the load-bearing capacity (Fig. S4l).

**Fig.3:**
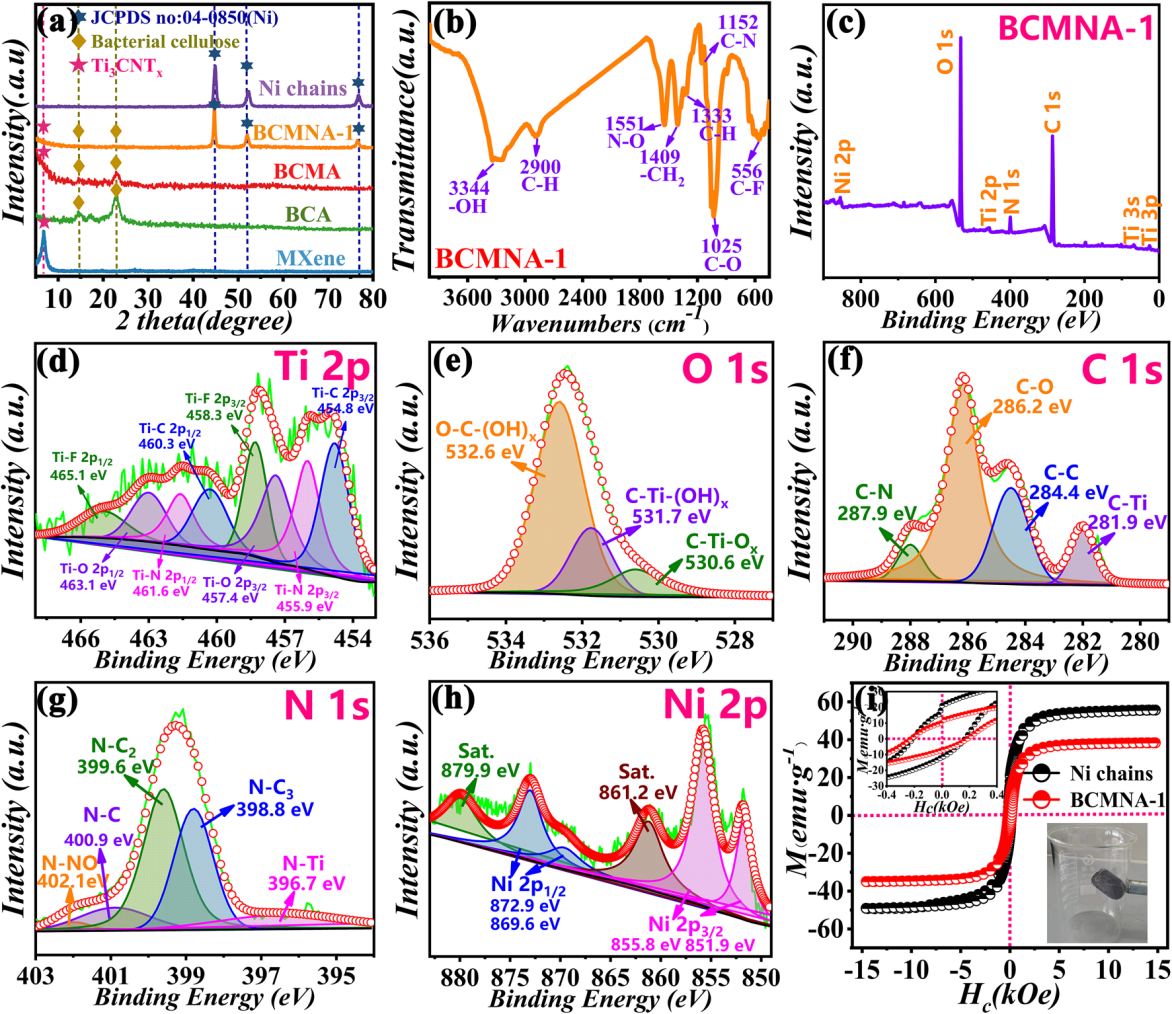
**a** XRD patterns. **b** FT-IR spectra, **c** wide-scan XPS survey of BCMNA-1 and corresponding high-resolution XPS survey: **d** Ti *2p,* **e** O 1*s*, **f** C 1*s*, **g** N 1*s*, and **h** Ni 2*p*; **i** room-temperature hysteresis loops of BCMNA-1 and Ni chains

Figure 3a shows the powder XRD patterns of the MXene, BCA, BCMA, BCMNA-1 and Ni chains. The characteristic peaks at 2*θ* = 14.8°, 16.7° and 22.8° are assigned to the (110), (110) and (200) planes of I-type cellulose crystals, and the corresponding interplanar spacing is 0.606, 0.527 and 0.390 nm, respectively. For the pure MXene, the single characteristic peak at 2θ = 7.0° associating with the (002) plane of typical MXene phase can be noticed [28], indicating the successful fabrication of MXene in this work. Furthermore, the characteristic peaks of NC and BCMNA at 2*θ* = 44.5°, 51.8° and 76.41°, corresponding to the (111), (200) and (220) planes, respectively, can be well identified and ascribed to Ni (JCPDS No. 04–0850). Thus, we can conclude that bacterial cellulose, MXenes and Ni chains exist in the BCMNA-1. The FT-IR spectra are used to investigate the structural composition or chemical group in bacterial cellulose and MXene (Fig. 3b). A sharp absorption peak located at 3344 cm^−1^ occurs due to the strong interaction of hydroxyl groups on the molecular chain of cellulose dextran through hydrogen bonds, resulting in the stretching vibration of -OH. The peaks at 2900 and 1333 cm^−1^ are attributed to the stretching vibration and bending vibration of C-H of the cellulose, respectively [29]. In addition, the three peaks at 1152 and 556 cm^−1^ indicating the vibrations of C–N and C–F, respectively, confirm the presence of functional groups of Ti_3_CNT_x_ in the BCMNA [30]. Peaks at 1550 cm^−1^ are attributed to vibrations of O-N [31]. Both BCA and BCMNA-1 exhibit the typical Raman mode peaks at 1465 and 1490 cm^−1^, corresponding to CH_2_ bending in cellulose molecules (Fig. S5). In comparison with BCA, the additional peaks of BCMNA-1 at 190, 450 and 600 cm^−1^ relate to Ti–O vibration and nonstoichiometric Ti-C vibrations on the surface of Ti_3_CNT_x_, which is consistent with the FT-IR spectra. This functional groups will cause dipole polarization when contracting with alternating EM field, representing favorable factors for the entrance and absorption of EMW in the samples.

The wide-scan survey spectrum of the XPS reveals that BCMNA-1 mainly consist of Ti, O, C, N and Ni elements (Fig. 3c). Coming into contact with the Ti 2*p*-related and O 1*s*-related spectrum, it can be concluded that abundant -OH, -F, -O groups exist on the surface of Ti_3_CNT_x_ (Fig. 3d, e) [32]. In the C 1*s* and N 1*s* spectrum, peaks located at 281.9 and 396.7 eV are connected with C-Ti and N-Ti bonds, respectively, indicating the successful fabrication of the Ti_3_CNT_x_ where Ti atoms are attached to C and N atoms (Fig. 3f, g) [30]. The Ni 2*p* peaks in Fig. 3h were currently performed to demonstrate the chemical states of Ni [33]. Furthermore, the accompanied satellite peaks imply the existence of a high-spin divalent state of Ni. The magnetic hysteresis loop of the samples measured by VSM that saturation magnetization (*M*_s_) value of BCMNA is 38.3 emu g^−1^, which is lower than that of NC (55.6 emu g^−1^) due to the presence of nonmagnetic components. The coercivity (*H*_c_) and remanent magnetization values (*M*_r_) of the BCMNA-1 and pure Ni chains are 213.2, 237.8 Oe and 7.1, 11.4 emu g^−1^, respectively. It shows that the BCMNA-1 exhibits a lower intrinsic coercivity when competing with NC, which is available for reinforcing the initial permeability, thereby improving the magnetic loss ability.

### EMW Absorption Performance

According to the transmission line equation, the essence of EMW absorption performance is to control the electromagnetic parameters. In order to clarify the requirements of electromagnetic parameters for ultra-low thickness absorbing materials, a series of exploratory calculations are given in Fig. S6. For a dielectric–magnetic loss coexisting-type absorber, it is complicated and unnecessary to sum up all the electromagnetic parameters corresponding to the characteristics due to the multivariable operation. Therefore, the method of fixed variables is used to explain the evolution of electromagnetic parameters in low thickness absorbing materials. Compared with the pure dielectric loss-type absorber, it can be found that the improvement of permeability via the introduction of magnetic loss media is beneficial to increase the absorption performance in low thickness. In addition, keeping permittivity larger at high frequencies is another key factor. Increasing dielectric blindly will often lead to the impedance mismatch. Therefore, this situation requires us to consider the microscopic mechanism, so that the absorber can maintain high EMW parameter and strong EMW loss ability at high frequencies.

In order to explore the associated EMW absorption performance, the complex permittivity (ε′ and ε″) and the complex permeability (μ′ and μ″) of the as-prepared samples with 15 wt% filling ratios are exhibited in Fig. S7 according to the transmission line theory. The real parts of the EMW parameters (ε′, µ′) represent the storage capability of EM energy, while the imaginary parts (ε″, µ″) connect with the attenuation ability of EM energy [34]. In the frequency range of 2–18 GHz, the ε′-f curve experiences an upward tendency, which could be ascribed to the lag phenomenon generated in the charges of absorber when responding the alternating EMW at high frequency [35]. As observed, BCA has the lowest ε′ value due to intrinsic inferior electrical conductivity. The ε′ values of BCA gradually increased after mixing with MXene and Ni chains, suggesting the strengthened electric energy storage properties. The increase of ε′ values with raising concentration of Ni chains suggests the dielectric promoting effect of highly oriented magnetic materials. The corresponding values of the ε′′ of these samples follow the order BCMNA-2 > BCMNA-1 > BCMA > BCA from 2 to 18 GHz, which shares the similar rank with ε′ values and accompanies with several relaxation peaks. The formation of resonance peaks in the ε″-f curve indicates the appearance of polarization relaxation, which could be further expressed by Cole–Cole semicircle based on Debye theory (Eq. S2) [36]. Each semicircle represents a polarization relaxation process and the radius of the semicircle indicates the strength of each process. As shown in Fig. S8, the number and radius of semicircles boost with the successive introduction of MXenes and Ni chains, prompting EMW in the absorbers to attenuate to the greatest extent. In addition to the interfacial polarization, the unique structure of aerogel also affects the fluctuation of electromagnetic parameters to a certain extent. Specifically, the measurement of electromagnetic parameters of absorber is aimed at heterogeneous composites, which is made up of absorbing agent and paraffin binder. Therefore, besides the physical properties of the absorbing agent itself, the dispersed state of the absorbing agent in paraffin wax also has a significant influence on the final characteristics of electromagnetic parameter. Compared with the traditional mixture between micro/nanopowder and paraffin, the state of electromagnetic response network generated by aerogel in paraffin will be different, which affects the trend of electromagnetic parameters changing with frequency. The alternative conductivity (σ) of samples can be associated with the ε′′, based on free-electron theory. Highly oriented Ni chains with conductive pathway in ultra-long range will boost the charge transport capacity at macroscale and make the conductivity loss capacity of aerogel to increase continuously with the concentration of magnetic medium. In particular, BCMNA-2 has a higher permittivity over 12 GHz in comparison with BCMNA-1, suggesting that the dielectric loss ability at high frequencies is improved when the concentration of Ni chains reaches a certain level. Compared with traditional magnetic materials distributed discretely in dielectric loss medium, the highly oriented Ni chains in this work can avoid being isolated by insulating medium when Ni and paraffin are mixed, which form conductive path inside the absorber, except MXenes, giving full play to the superiority of one-dimensional structure and multidimensional hybrid characteristics. Figure S7c-d displays the µ′-f and the µ″-f curve of samples. Briefly, the BCA fails to induce magnetic loss because of its nonmagnetic characteristic, where the values of μ′ and μ″ fluctuate near 1 and 0, respectively. The µ′ and µ″ values of the magnetic samples (BCMNA-1 and BCMNA-2) are higher than those of the nonmagnetic samples (BCMA and BCA) resulted from the ferromagnetic properties of Ni chains. It should be noted that the magnetic energy radiated from the induced magnetic field of magnetic absorber will be converted into electrical energy, bringing about negative μ″ value of BCMNA-1 and BCMNA-2 at high frequency range.

**Fig. 4:**
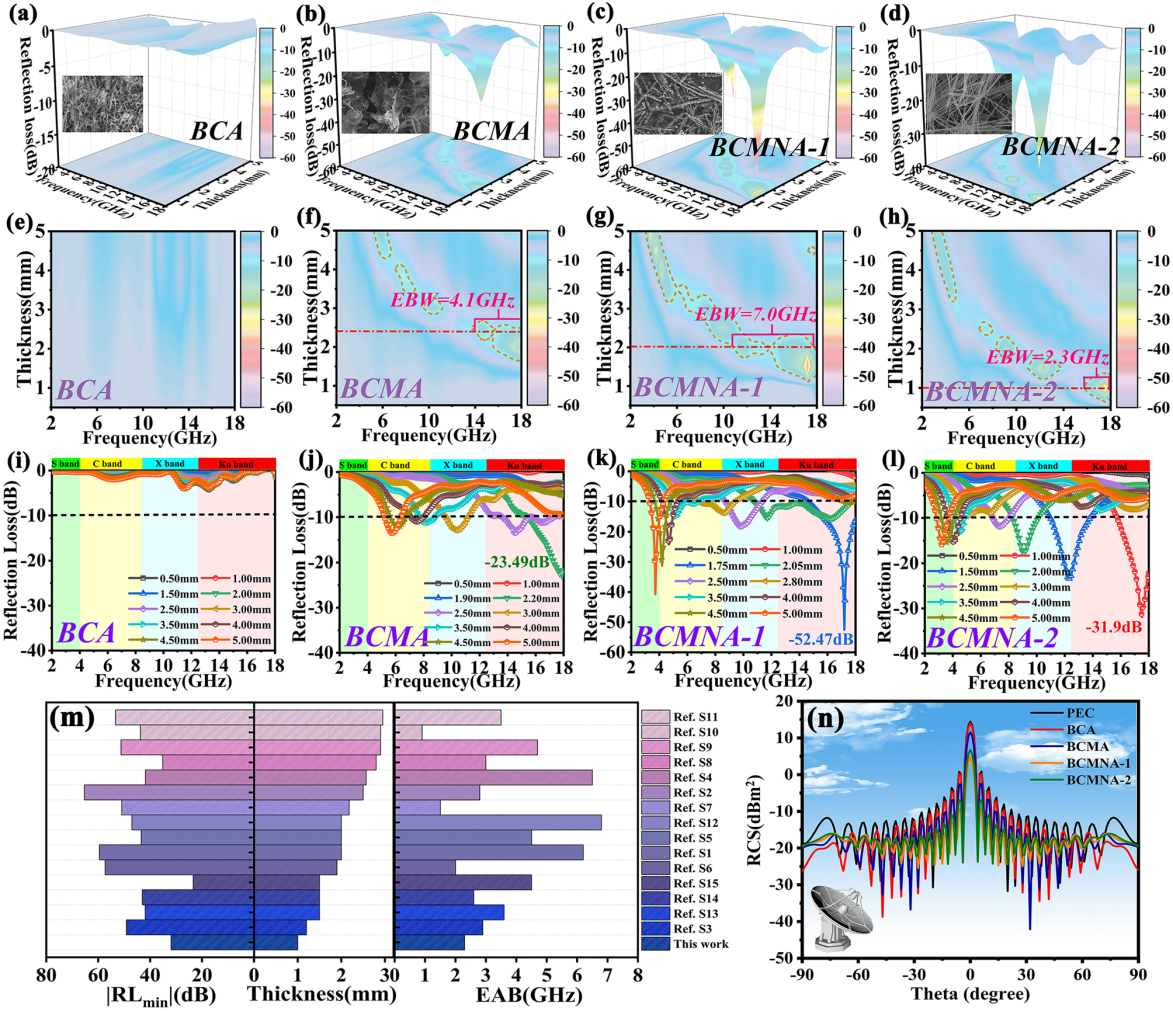
3D RLs-f curves, corresponding contour map and 2D *RLs-f* curves of **a**, **e**, **i** BCA, **b**, **f**, **j** BCMA, **c**, **g**, **k** BCMNA-1, and **d**, **h**, **l** BCMNA-2 with 15 wt% filling ratios under 0.5–5.0 mm; **m** comparison image of similar reported EMW absorbing materials; **n** RCS simulated curves of samples-PEC composites and pure PEC with scanning angles from  − 90° to 90°

**Fig. 5:**
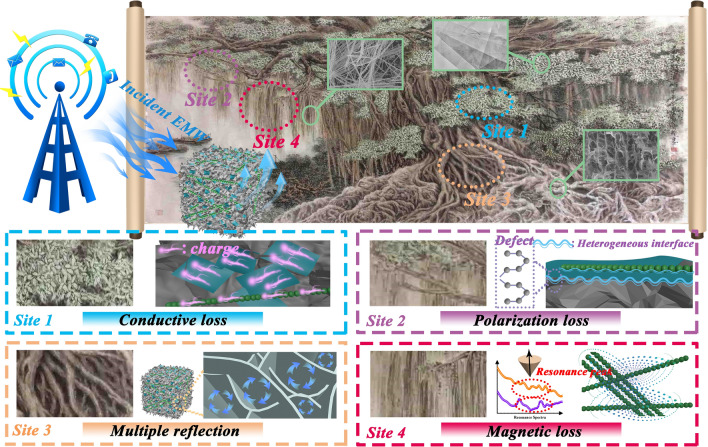
Schematic illustration of EMW absorption mechanisms for the BCMNA with different strategies: site 1) progressive conductive loss network; site 2) multicomponent-induced polarization loss; site 3) porous-derived multiple scattering; site 4) macroscopic scale-enhanced electromagnetic cooperation; and the insert ink painting derive from Chinese painter Yunlong Feng’s “Banyan Tree”

The EMW performance is currently concerned on two aspects: the strong minimum reflection loss (*RL*_min_) and wide effective bandwidth (EBW, *RL* ≤  − 10 dB). The related calculation formula is [37, 38]:

$$RL=20log\left|\frac{{Z}_{in}-{Z}_{0}}{{Z}_{in}+{Z}_{0}}\right|$$

where Z_in_ and Z_0_ are input impedance and free space impedance, calculated based on Eq. S1. Figure 5a–l shows the 3D *RL-f* curves and 2D *RL-f* curve of BCA, BCMA, BCMNA-1 and BCMNA-2 with 15 wt% filling ratio under 0.5–5 mm. The *RL*_min_ of BCA is merely − 4 dB at 4.0 mm (Fig. 4a). The corresponding 2D *RL-f* curve also shows that there are no EBW absorbing areas, implying the poor EMW absorbing properties of BCA due to ultra-low intrinsic conductivity. Even though the mixing with MXene brings about the strengthen of dielectric loss, the obtained BCMA demonstrates the limited improvement of EMW absorption performance resulted from the lack of magnetic loss ability. The *RL*_min_ value of BCMA is − 24.6 dB at 17.8 GHz with 2 mm thickness. Upon the thickness rising to 2.5 mm, its EBW can almost cover the entire Ku band (4.7 GHz) (Fig. 4b, f, j). Owing to the regulation of component and design of structure, the obtained ficus microcarpa-like aerogel demonstrated remarkable EMW absorption performance (Fig. 4c, g, k) with the strongest *RL*_min_ of − 52.5 dB at 1.75 mm thickness, and a wide EBW of 7 GHz (from 10.7 to 17.7 GHz) at 2.05 mm thickness. Compared with BCA and BCMA, the *RL*_min_ of BCMNA-1 is improved by 13.1 and 2.2 times, respectively. Furthermore, by tailoring the matching thickness from 0.5 to 5.0 mm, the *RL-f* curve lower than − 10 dB can be accomplished in the scope of 2–18 GHz, covering the whole C, X and Ku band. The variation of these curves is also related to the resonant absorption generated by the quarter-wavelength model, where the reflected EMW is totally offset at the absorber–air interface because of the 180 ° phase difference between the incident and reflected EMW. The results demonstrate that the enhanced RL value and broadened EBW can be achieved upon the guidance of electromagnetic cooperation. The combination of dielectric materials and magnetic component, by means of their cooperative effect, not only rises the permittivity and permeability of pure dielectric materials according to the results of the above EM parameters, but also solves the surface skin effect of pure magnetic materials, increasing the response and effect between absorbers and alternating EMW. Furthermore, BCMNA-2 with highest conductivity and magnetic component concentration is taken advantage of verifying the preponderance of electromagnetic cooperation in macroscopic scale (Fig. 4d, h, l). The *RL*_min_ value of − 31.9 dB can be obtained at 17.52 GHz with 1 mm thickness and its EAB also achieves 2.3 GHz, which is unique among other dielectric-magnetic loss coexisting-type absorbers. According to the aforementioned analysis of electromagnetic parameters, BCMNA-2 still maintains a high permittivity at high frequencies, which is the main reason for its EMW absorbing performance under thin thickness and consistent with the result of deduction. In order to further reveal the superiority of electromagnetic cooperation at macroscopic scale, Fig. S9 shows the *RL-f* curves of the samples in S band and Ku band at optimum thickness. In the Ku band, the *RL* value in all frequency range of BCA is higher than − 10 dB, indicating its negligible EMW absorption performance. In comparison, with the inducing of MXenes and Ni chains, the optimal *RL*_min_ value of BCMA and BCMNA-1 reaches − 22.6 and − 52.5 dB, and the EAB broadens to 2.8 and 3.5 GHz, respectively. When the concentration of Ni chains increases, the optimal *RL*_min_ value and EAB show a decreased tendency, but the matching thickness improves to 1.00 mm. This evolvement rule reveals that electromagnetic cooperation at macroscopic scale can effectively optimize the matching thickness of Ku band. In S band, the *RL* value of BCA and BCMA is higher than -10 dB, accompanied with no EBW absorbing areas. In comparison with magnetic samples at the same thickness of 5 mm, the *RL*_min_ value of BCMNA-1 and BCMNA-2 reaches − 40.7 and − 15.8 dB, and the EBW widens to 1.0 and 0.8 GHz. As a consequence, the EMW response performance (*RL*_min_ and EBW) of samples in Ku band and S band can be manipulated by introducing of MXene and Ni chains, respectively, which establishes the significant position of electromagnetic cooperation for amelioration of the multifrequency EMW absorption trait. Meanwhile, compared with the similar works (Fig. 4m and Table S1), the as-prepared BCMNA-2 has the ability of attenuating EMW even at ultra-low thickness (1 mm), which is superior to similar works. Although the reflection intensity and EAB are slightly inferior, we point out the excellent results in matching thickness, which achieves the core idea about the construction of EMW absorber with ultra-low thickness in this work. To further evaluate the EMW absorption performance under practical application conditions, the RCS distributions of as-prepared samples with similar matching thickness and filling ratio on perfect electric conductor (PEC) plate are simulated by the CST software (Fig. S10) [39]. Figure 4n shows 2D projections of the RCS distributions of the samples- PEC and the pure PEC plane at 11 GHz (X band radar) from − 90° to 90°, and the positive X axis is chosen as the incident direction while the Z-O-Y plane is selected as the mapping plane. Obviously, as compared to pure PEC, BCA-PEC and BCMA-PEC, the PEC plate coated with 2 mm BCMNA-1 and BCMNA-2 layer effectively suppresses the EMW scattering from − 30° to 30° with several fluctuations, which corresponds accurately with the EMW absorbing property calculated by transmission line theory. In particular, the maximum RCS reduction values reach 9.89 dB m^2^ for electromagnetic cooperative sample when the theta locates at 0 °, indicating excellent radar wave attenuation ability without the assist of PEC.

### EMW Absorption Mechanisms

Inspired by the biological morphology in nature, the involved EMW loss mechanism of BCMNA is illustrated by means of Chinese ink painting of a ficus macrocarpa (Yunlong Feng’s “Banyan Tree”), which can be divided into the indicated components (Fig. 5).

Progressive conductive loss network: inheriting the framework of bacterial cellulose–chitosan, the obtaining of BCMA effectively avoids the stacking of several few-layered MXene lamellas and comes into being a 3D current conduction network where the MXene lamellas are bridged to each other. As EMW penetrates the network, the excited charges on the MXene migrate along the lamellar direction or hop to other neighbored lamellas, leading to multidirectional flow of charge inside the aerogel. This multidirectional conduction network provides a positive transmission pathway for the migrator of the free charges and converts the electric energy to the thermal or other formal energy because of the resistance of the MXene, thereby remarkably promoting the dielectric loss property (Fig. 5, Site 1) [40]. Moreover, because of the high concentration of free electrons inside the transition metal, the conductivity loss ability of aerogel is improved progressively with the introduction of Ni chains [41]. In particular, the electrical conductivity of oriented chains is inferior to that of the random distribution ones because direct contact between Ni nanoparticles replaces the original empty space with high resistivity. Compared with the crystalline 1D magnetic samples reported in other work, the as-synthesized Ni chains has a higher permittivity because the highly oriented structure provides more unhindered transmission channel for the electrons [42]. In addition, the conductivity also dominates the attenuation constant (*α*) and impedance matching, which should be taken into consideration for EMW absorption performance [43]. The former one stands for the dissipated ability of incident EMW energy by multiple mechanisms and the latter one represents the special boundary conditions that minimize the reflectivity of EMW on the surface of the medium, resulting in as much EMW inside absorber as possible. The *α*-f curve and delta model illustrated in Fig. S11 evidently show that the *α* and the impedance matching of BCMNA is progressively improved after the introduction of MXene and Ni chains. Connecting with the ε′-f and ε″-f curves as displayed in Fig. S7a-b, the BCA and NC identically deliver the lowest and the highest α values in comparison of other samples over the entire tested frequencies, ensuring the premise for obtaining poor or high EMW absorption performance. The super-high conduction loss in pure metal chains results from rapid migration of high concentration internal charges that should responsible for the boosted *α* values [44]. Besides, based on previous reports, the delta value (Δ) closed to zero (|Δ|< 0.4) indicates a satisfied impedance matching degree of samples [45, 46]. As illustrated, the BCMNA-1 and BCMNA-2 have a broader Δ values than BCA and BCMA, implying the improved impedance matching properties via regulating the conductivity through compounding (Fig. S12). Considering these two factors, we can conclude that progressive conduction loss network determines the promoted α values as well as the optimized Δ degree simultaneously, further dominating the final EMW performance.

Multicomponent-induced polarization loss: As evidenced in Fig. S8, the Cole–Cole curves of BCMNA possess plentiful semicircles in the measured frequency range and contribute to suitable frequency dispersion and dielectric loss ability, bringing about the high-intensity and wide-band EMW absorption. Generally, the interfacial polarization and associated relaxation caused by massive heterogeneous interfaces on samples are responsible for the appearance of semicircles [47]. The numbers of semicircles are ascendant following the order of BC < BCMA < BCMNA, suggesting that the polarization capacity increases from single component to multicomponent [19, 48]. The aforementioned SEM morphology illustrated that the bacterial cellulose–chitosan dispersion is completely coating the MXene lamellas and the Ni chains in the freeze-drying process, boosting the expansion of the contacting area as well as the intensity of interfacial polarization. In such ficus microcarpa-like microstructure, there are three types of heterogeneous interface: (1) bacterial cellulose–MXene interfaces, (2) bacterial cellulose–Ni chains interfaces and (3) MXene–Ni chains (Fig. 5, Site 2). Among these heterostructures, the positive charges locate on the surfaces of bacterial cellulose or MXene while the negative charges move to the Ni chains side on account of the relatively high dielectric characteristics and electrical conductivity of Ni, thereby generating a dipole electric field under the reaction of alternating EM field. The EMW energy is dissipated during the polarization process and relaxes to the dynamic equilibrium via the contact of the bacterial cellulose–MXene-Ni heterojunction. In addition to the interfacial polarization between different components, the molecular polarization formed upon each component also promotes the polarization loss to a certain extent. Usually, molecules (polar/nonpolar) have inherent dipole moments. When an external EM field is applied, the intrinsic dipole moments of the molecules will rearrange with the direction of the EM field, resulting in the total vectors sum of the dipole moments away from zero, and molecular polarization appears [49, 50]. Polarization center, including carboxyl groups on bacterial cellulose, functional groups such as O and F on the MXene, and surface defects of each group, gives rise to polarization relaxation and related molecules polarization also intensify the EMW performance [51, 52].

Porous-derived multiple scattering: Notably, the unique porous structure of aerogel scatters repeatedly the propagated EMW, which provides enough sites for the absorption and exhaustion of EMW (Fig. 5, Site 3) [53]. In other words, the air cavities play as dihedral angles to extend the propagation path of EMW and assist the reflection of internal EMW. According to the Maxwell–Garnett theory, the permittivity of samples can be regulated by the variation of volume ratio between air medium and solid medium inside the material, ensuring the penetration of EMW into the lossy component with lower impedance gap and dramatically facilitating the transform of EMW energy into thermal energy [54]. Moreover, the internal porous structure not only lightens the density compared with solid counterpart but also enlarges the specific surface area, thereby providing more space for modification of MXene and Ni chains, as well as enhancing the polarization loss.

**Fig. 6:**
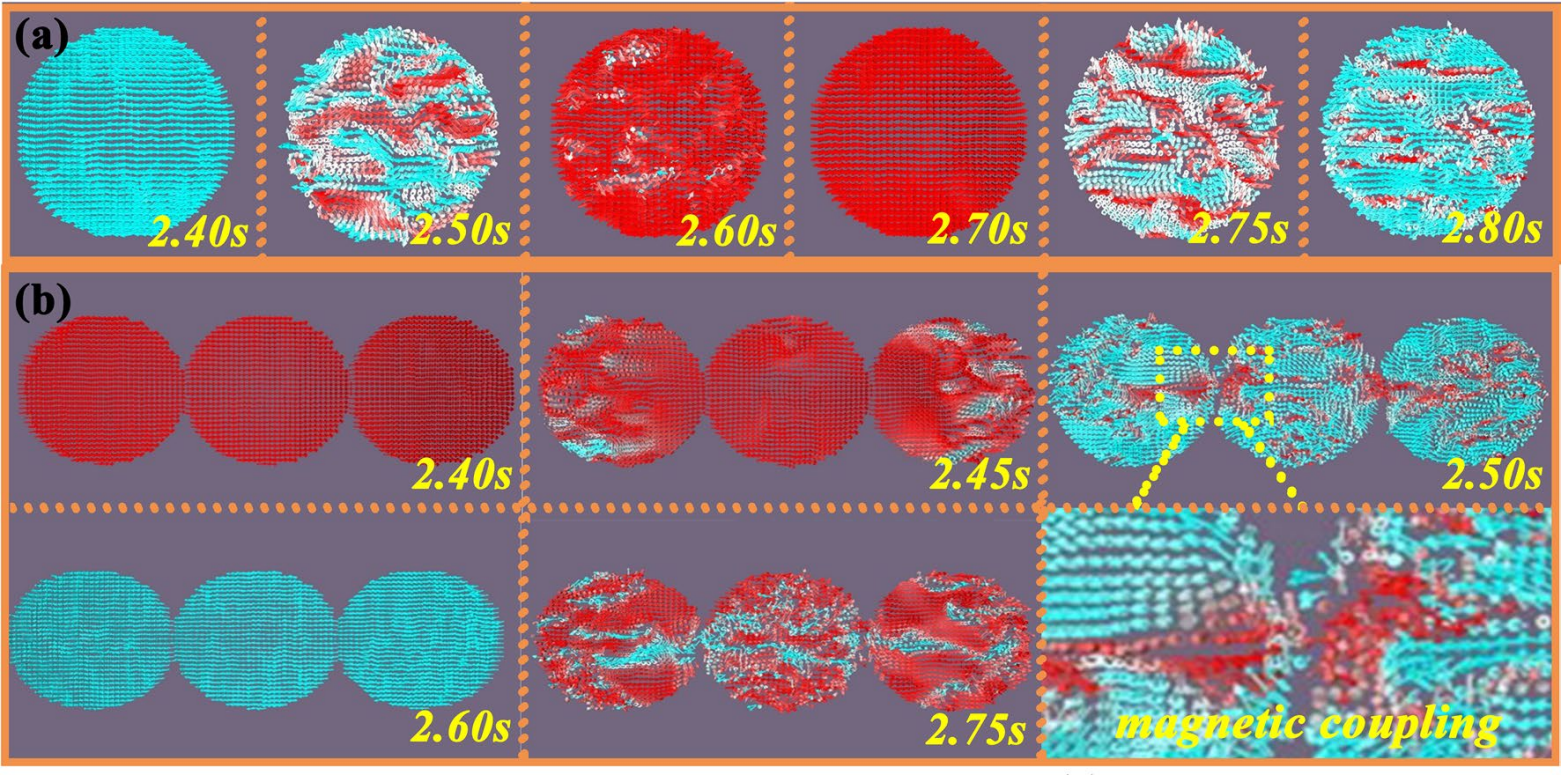
Micromagnetic simulation result of **a** single Ni particle and **b** Ni chain

Macroscopic scale-enhanced electromagnetic cooperation: In general, magnetic resonance and eddy current loss play a major role in the magnetic loss at the tested range (Fig. 5, Site 4) [55]. For BCMNA, the assembled Ni chains acts as a ferromagnetic medium and have a magnetic response under the alternating electromagnetic fields. In the frequency range, fluctuating C_0_-f represents that the natural and exchange resonances occur simultaneously at high or low frequencies in addition to eddy current loss (Fig. S13) [56]. More importantly, with the improvement of dimension, one-dimensional (1D) highly oriented Ni chains have stronger electromagnetic coupling than common zero-dimensional (0D) magnetic nanoparticles, which can be explained from the aspects of saturation magnetization, domain motion and magnetic flux line. First of all, for the 1D chain structure composed of regular magnetic nanoparticles, it can be regarded as a rodlike magnetic medium, which shows a smaller demagnetization factor than the dispersed nanoparticles under the external magnetic field. The decrease of demagnetization factor results in the increase of crystallinity and saturation magnetization, which leads to the increase of *M*_s_ value and enhanced magnetic loss ability [57]. Secondly, differed from the fixed domain of the 0D magnetic nanoparticles, the domain motion starts to happen in response to the alternating electromagnetic field, leading to the characteristic anisotropy of the magnetic chains and improved the ability of magnetic energy storage. In order to clarify this phenomenon clearly, micromagnetic simulation by Mumax3 software at different frequencies is applied, as shown in Fig. 6a, b and S14-S17. Owing to the changes in the phase of magnetic fields, typical magnetic vortex generates, migrates and disappears frequently inside the region of single Ni particle, relating to the magnetic loss mechanism of domain wall migration. As the frequency increased from 2 to 18 GHz, the vortex response and magnetic domain reversal rate are accelerated due to the enhanced mutual effect between the local demagnetizing field and the external magnetic field (Movie S1). When the Ni particles are assembled into the chains, obvious magnetic coupling phenomenon can be discovered in the contacted area between particles, where stray magnetic field keeps a similar dynamic change and mutual induction process come into being [58]. Therefore, the domain changes of a single magnetic particle affect the changes of adjacent magnetic particles, leading to the propagation of domain motion along the chain and the generation of dense magnetic coupling network (Movie S2). The as-prepared highly oriented Ni chains in this work deeply strengthen this propagation state due to the increased path, boosting the optimization of magnetic loss properties. Thirdly, during the process of chains formation, the magnetic flux changes from the scattered distribution to annular distribution. Two different regions in each chain are coupled to form a semicircular magnetic flux line, and the coupling interaction between different chains also changes the distribution orientation of magnetic flux lines. As the length of chain increases, the coupling interaction becomes stronger, strengthening the magnetic loss as well. Importantly, the Ni chains prepared in this work are ~ 1 cm long, through a strong external magnetic field, superior to all previous work related to 1D magnetic analogs. The magnetic coupling between highly oriented Ni chains and MXene at macroscale constitutes an electromagnetic cooperative loss network with continuous conduction over an ultra-long distance. With the increased concentration of the magnetic medium, the reinforced cooperative network leads to high permittivity at high frequency, thereby achieving exclamatory EMW absorption at ultra-low thickness.

## Conclusions

Overall, in order to achieve ultrathin matching thickness via electromagnetic cooperation at a macroscopic scale, a highly directional self-assembly engineering derived ficus microcarpa-like aerogel was prepared under the driving force of temperature gradient and magnetic field. The highly oriented Ni chains with macroscopic morphology (~ 1 cm in length) exhibit strong anisotropy and strong magnetic coupling, which was confirmed by micromagnetic simulation. The Ti_3_CNT_x_ lamellas, wrapped in the bacterial cellulose-based skeleton, generate an interlaced and persistent conductive network and the decorated Ni chains with multiple magnetic coupling types scatter all over the aerogel. Due to the macroscopic scale-enhanced electromagnetic cooperation, the magnetic aerogel exhibits an outstanding EMW performance with a *RL*_min_ of − 31.9 dB at an ultrathin thickness of 1 mm. Electromagnetic cooperation at macroscale enables the aerogel to generate an uninterrupted dielectric–magnetic loss network in paraffin wax, resulting in strong permittivity at high frequency range. The RCS simulation result further explains the promoting effect of electromagnetic cooperation on EMW attenuation. Thus, this work not only provides a bright pathway for achieving magnetic material at centimeter scale with highly directional self-assembly method, but also reveals the macroscopic scale-enhanced electromagnetic cooperative strategy toward high-efficient EMW absorber.

## Supplementary Information

Below is the link to the electronic supplementary material.

Supplementary file1 (GIF 4705 kb)

Supplementary file2 (GIF 8094 kb)

Supplementary file3 (PDF 2192 kb)
