# Supplementary figures and images for: Macroscopic Electromagnetic Cooperative Network-Enhanced MXene/Ni Chains Aerogel-Based Microwave Absorber with Ultra-Low Matching Thickness
Source: Nanomicro Lett. 2022 Jul 5;14:140. doi: 10.1007/s40820-022-00869-7 (PMC9256896; doi:10.1007/s40820-022-00869-7)

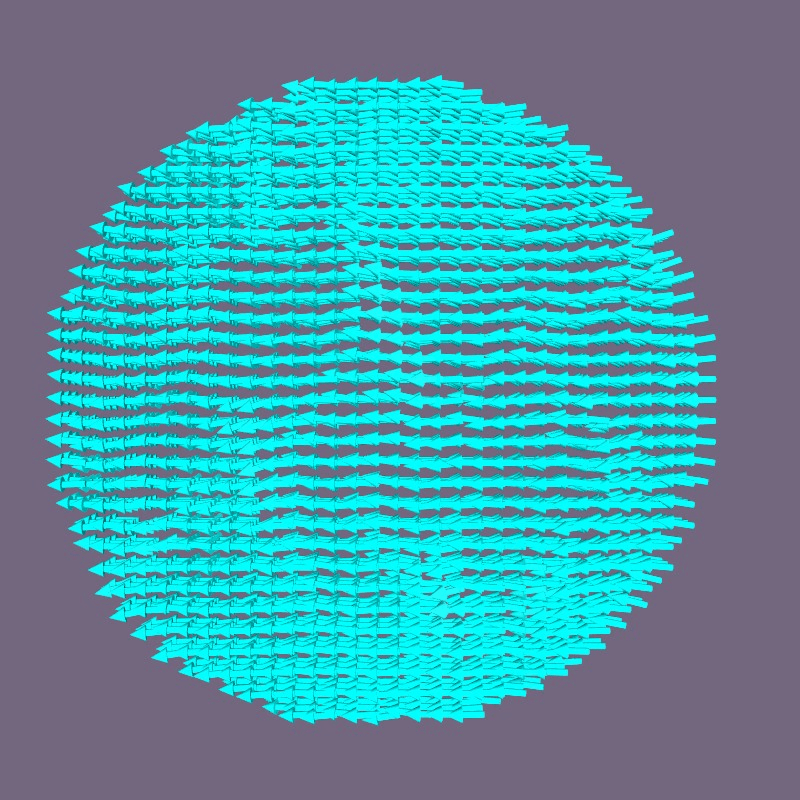

Supplement: Supplementary file 1 — Supplementary file1 (GIF 4705 kb) [file 40820_2022_869_MOESM1_ESM.gif]

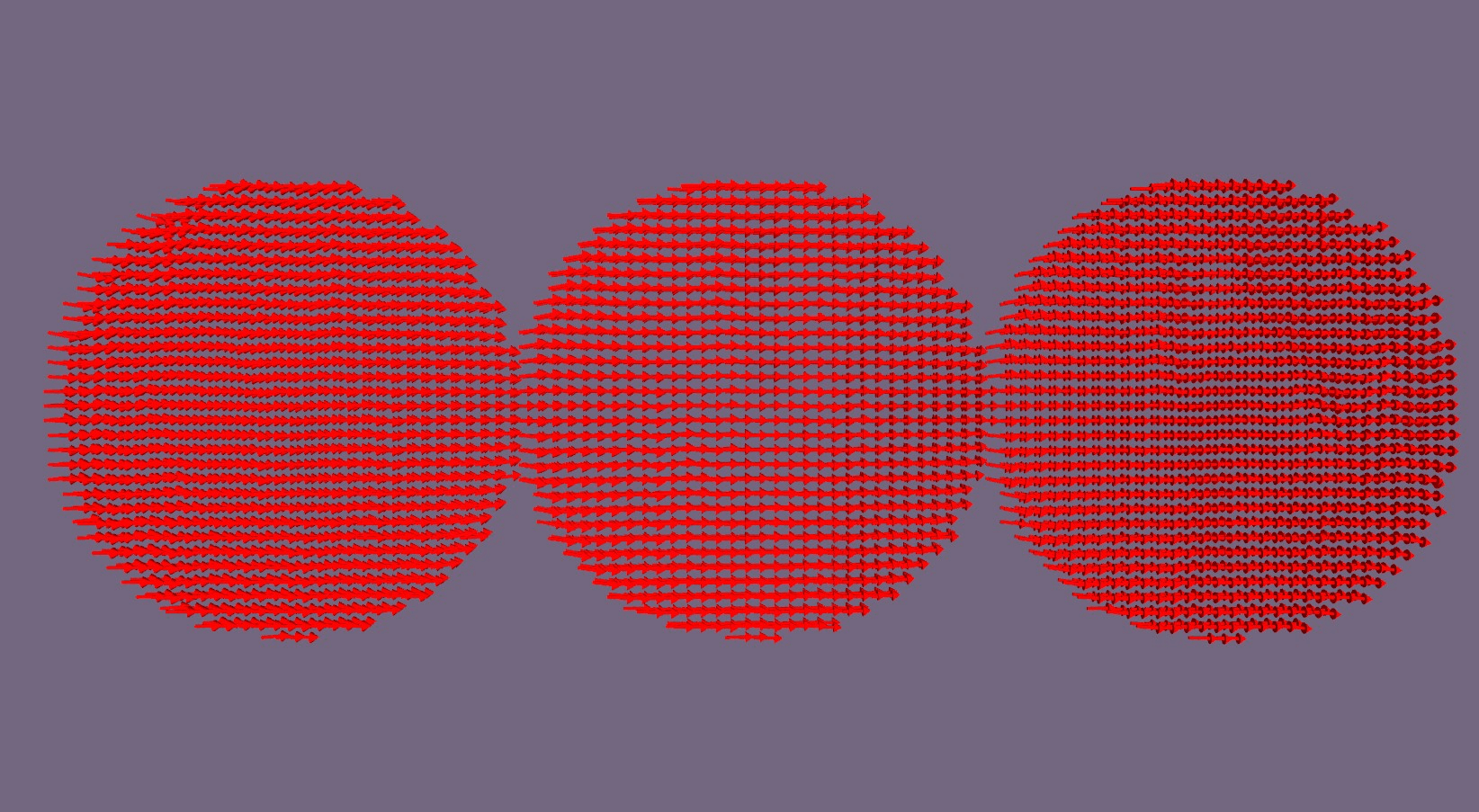

Supplement: Supplementary file 2 — Supplementary file2 (GIF 8094 kb) [file 40820_2022_869_MOESM2_ESM.gif]
