# Supplementary material for: Macroscopic Electromagnetic Cooperative Network-Enhanced MXene/Ni Chains Aerogel-Based Microwave Absorber with Ultra-Low Matching Thickness
Source: Nanomicro Lett. 2022 Jul 5;14:140. doi: 10.1007/s40820-022-00869-7 (PMC9256896; doi:10.1007/s40820-022-00869-7)
Supplement: Supplementary file 3 — Supplementary file3 (PDF 2192 kb) [file 40820_2022_869_MOESM3_ESM.pdf]

Supporting Information for

Macroscopic Electromagnetic Cooperative Network Enhanced MXene/Ni Chains Aerogel-Based Microwave Absorber with Ultra-Low Matching Thickness

Fei Pan[1], Yanping Rao[2], Dan Batalu[3], Lei Cai[1], Yanyan Dong[1], Xiaojie Zhu[1], Yuyang Shi[1], Zhong Shi[2], Yaowen Liu[2], Wei Lu[1,] *

[1] Shanghai Key Lab. of D&A for Metal-Functional Materials, School of Materials Science & Engineering, Tongji University, Shanghai 201804, P. R. China

[2] Shanghai Key Laboratory of Special Artificial Microstructure Materials and Technology, School of Physics Science and Engineering, Tongji University, Shanghai 201804, P. R. China

[3] Materials Science and Engineering Faculty, Politehnica University of Bucharest, Bucharest, 060042, Romania

*Corresponding author. E-mail: weilu@tongji.edu.cn (Wei Lu)

S1 RCS Simulation

The RCS simulation of EM absorber was used CST software based on far-field response. In this simulation, the constructed model consists of the absorber/ paraffin layer and the perfect electric conductor (PEC) layer, where PEC also is also regarded as a reference value to determine the RCS reduction. In detail, the length and width of each layer were set as 200 mm and the thicknesses of the absorber/paraffin layer (15% filling ratio) and the PEC layer were set as 2.0 and 5.0 mm, respectively. The simulation used plane wave excitation, and the EMW propagates in the negative direction of the x-axis and the electric polarization direction is along the z-axis. In addition, the free space boundary conditions were used and the center frequency was defined as 11 GHz. The RCS value of the simulated FFSC uses the time domain method for calculation and the detail equation expressed as following:

$$\sigma(dB\, m^2) = 10log\left[\frac{4\pi S}{\lambda^2}\left|\frac{E_s}{E_i}\right|^2\right]$$

where S is the area of the layer, λ is the length of the incident EMW, E_s and E_i are the electric field intensity of transmitting waves and receiving waves, respectively.

S2 Micromagnetic Simulation

The micromagnetic simulation in the paper is carried out by the Mumax3 software. It is an open-source software and inputted by a subset of Go's syntax. The dynamic physical process is simulated on the basis of the Landau-Lifshitz-Gilbert equations and the principle of minimum energy. The solving process of 3D model is relied on the finite difference algorithm.

In this work, two kinds of simulation program are carried on to describe the dynamic spin structure of individual Ni nanoparticle and Ni nanoparticle array, respectively. For the individual nanoparticle, the universe is 500 × 500 × 500 nm. The particle is consisted of several grains with random shape and orientation. For simulating the Ni chain, the universe is 500 × 500 × 1500 nm. The gap between neighbor particles is zero. The magnetic parameters of the Ni nanoparticle are shown below: saturation magnetization $M_s = 1.2\times10^6$ A m^{-1}, micromagnetic exchange constant $A = 1\times10^{-12}$ J/m, magnetocrystalline anisotropy constant $Ku_1 = 1.15\times10^5$ J/m^3, the anisotropic direction is vector (0, 0, 1), the magnetic field direction is Uniform (0, 0,

1) and damping factor of spin precession α = 0.15. The software Muview is used to visualize the recorded data. The frequencies of applied field in the simulation are 2, 10, and 18 GHz.

S3 Corresponding Formula in Manuscript

Formula S1: Reflection loss

$$Z_{in} = Z_0\left(\frac{\mu_r}{\varepsilon_r}\right)^{1/2}\tanh[j(2\pi fd/c)(\varepsilon_r\mu_r)^{1/2}]$$

$$RL = 20\log|(Z_{in}-Z_0)/(Z_{in}+Z_0)|$$

Z_0 stands for the impedance of free space, Z_{in} is the input impedance of the absorber, d is the thickness of the absorber, and c represents the velocity of light.

Formula S2: Cole-Cole semicircle

$$\varepsilon_r = \varepsilon' - j\varepsilon'' = \varepsilon_\infty + \frac{\varepsilon_s - \varepsilon_\infty}{1 + j2\pi f\tau}$$

$$\varepsilon' = \varepsilon_\infty + \frac{\varepsilon_s - \varepsilon_\infty}{1 + (2\pi f\tau)^2}$$

$$\varepsilon'' = \varepsilon_\infty + \frac{2\pi f\tau(\varepsilon_s - \varepsilon_\infty)}{1 + (2\pi f\tau)^2}$$

$$\left(\varepsilon' - \frac{\varepsilon_s + \varepsilon_\infty}{2}\right) + (\varepsilon'')^2 = \left(\frac{\varepsilon_s - \varepsilon_\infty}{2}\right)^2$$

ε_s, ε_∞, f and τ stands for the static dielectric constant, the dielectric constant at infinite frequency, the frequency and the polarization relaxation time.

Formula S3: attenuation constant

$$\alpha = \frac{\sqrt{2}}{c}\pi f\sqrt{(\varepsilon''\mu'' - \varepsilon'\mu') + \sqrt{(\varepsilon''\mu'' - \varepsilon'\mu')^2 - (\varepsilon''\mu' + \varepsilon'\mu'')^2}}$$

Formula S4: calculated delta value

$$|\triangle| = \left|\sinh^2(Kfd) - M\right|$$

$$K = \frac{4\pi\sqrt{\mu'\varepsilon'} \times \sin\left(\frac{\delta_e + \delta_m}{2}\right)}{c \times \cos\delta_e \times \cos\delta_m}$$

$$M = \frac{4\mu'\cos\delta_e \times \varepsilon'\cos\delta_m}{(\mu'\cos\delta_e - \varepsilon'\cos\delta_m)^2 + \left[\tan\left(\frac{\delta_{m-\delta_e}}{2}\right)\right]^2 \times (\mu'\cos\delta_e + \varepsilon'\cos\delta_m)^2}$$

Formula S5: eddy current factor

$$C_0 = \mu''(\mu')^{-2}f^{-1} = 2\pi\mu_0 d^2\sigma/3$$

μ_0 and σ are the permeability of vacuum and the electric conductivity, respectively.

Formula S6: quarter-wavelength model

$$t_m = nc/(4f_m\sqrt{|\mu_r||\varepsilon_r|}) \quad (n=1, 3, 5\&)$$

$f_m$is the peak frequency, and t_m is the absorber thickness.

S4 Supplementary Figures and Table

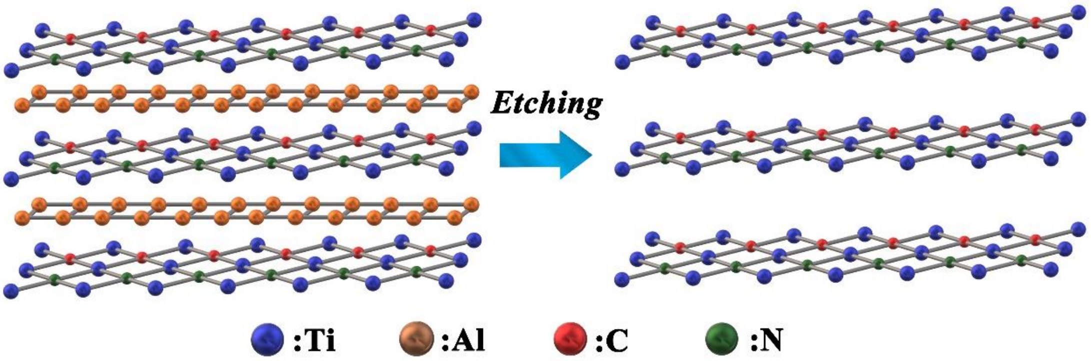

Fig. S1 Crystal structures of Ti_3AlCN and Ti_3CN nanosheets

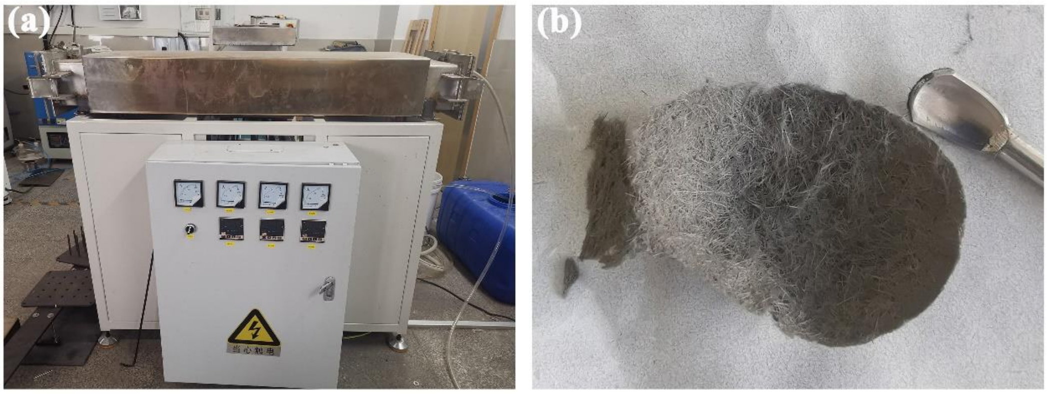

Fig. S2 (**a**) digital image of novel magnetic heat treatment furnace; (**b**) digital image of highly oriented Ni chains

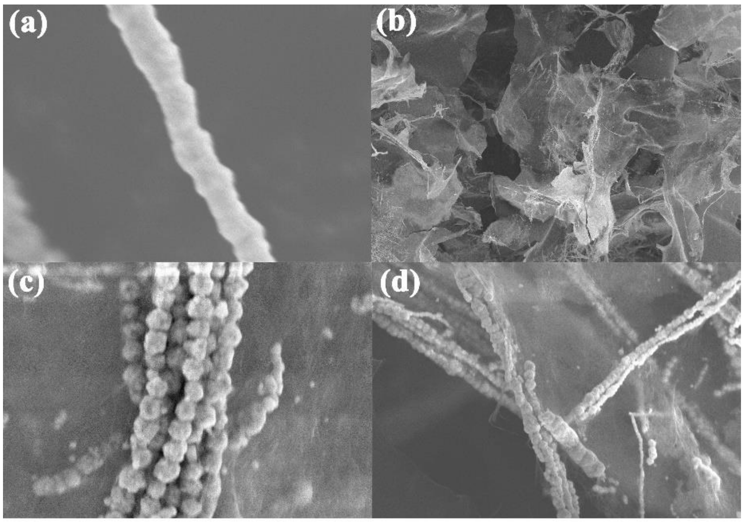

Fig. S3 SEM images of (**a**) Ni chains, and (**b-d**) BCMNA-2

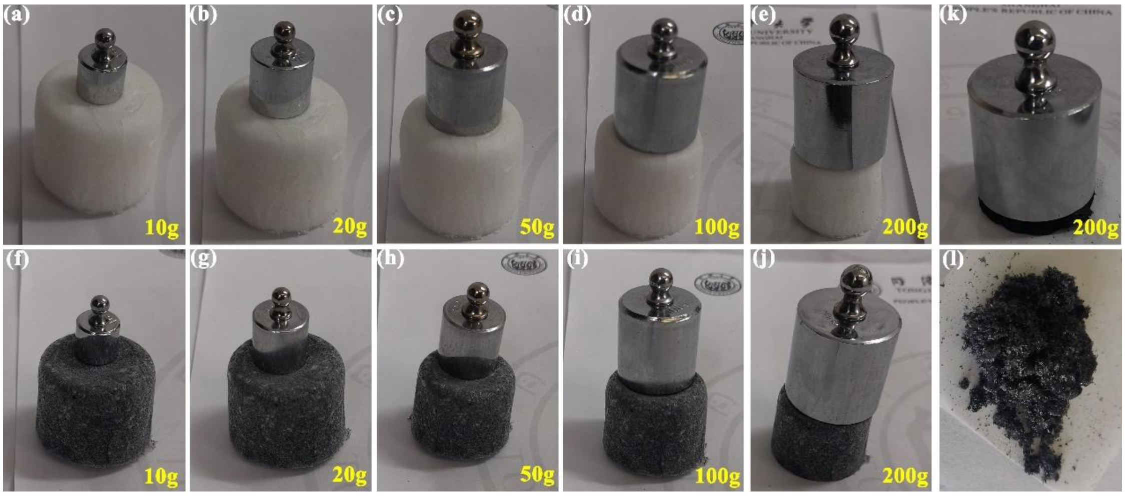

Fig. S4 Load-bearing capacity of (**a-e**) BCA and (**f-j**) BCMNA-1 with 10/20/50/100/200g weight; (**k**) load-bearing capacity of BCMNA-1 (without adding chitosan) with 200g weight; (**l**) digital image of pure Ti_3CNT_x aerogel

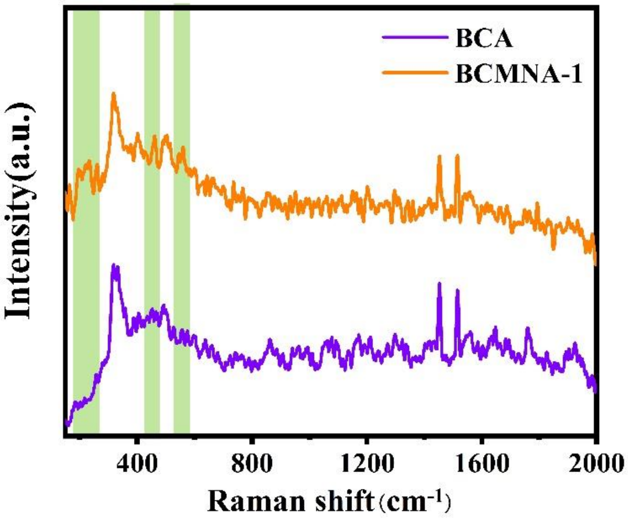

Fig. S5 Raman spectra of BCA and BCMNA-1

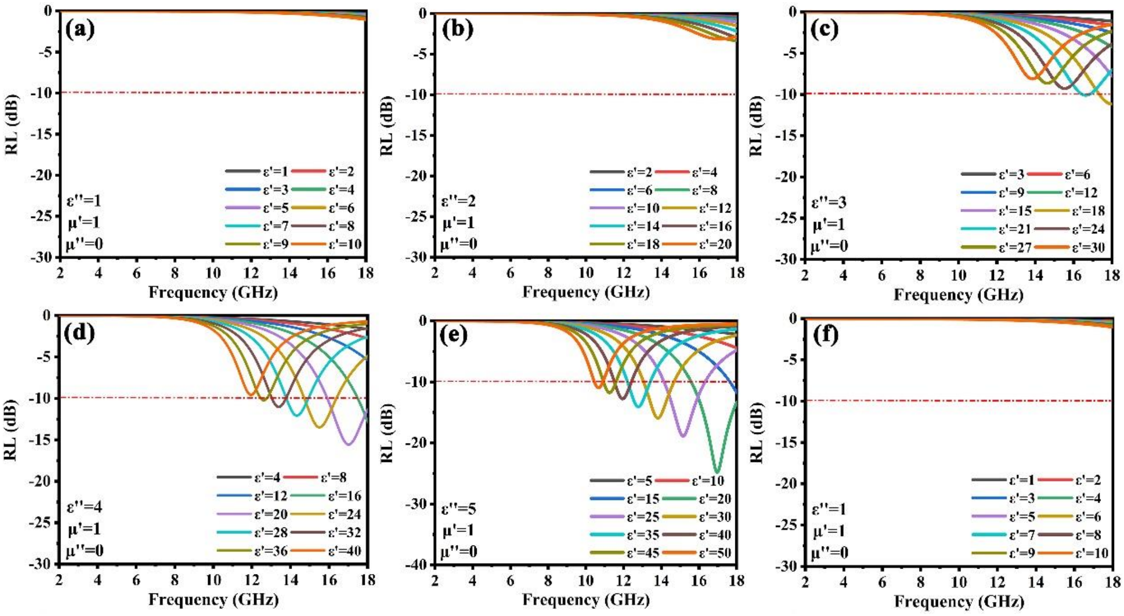

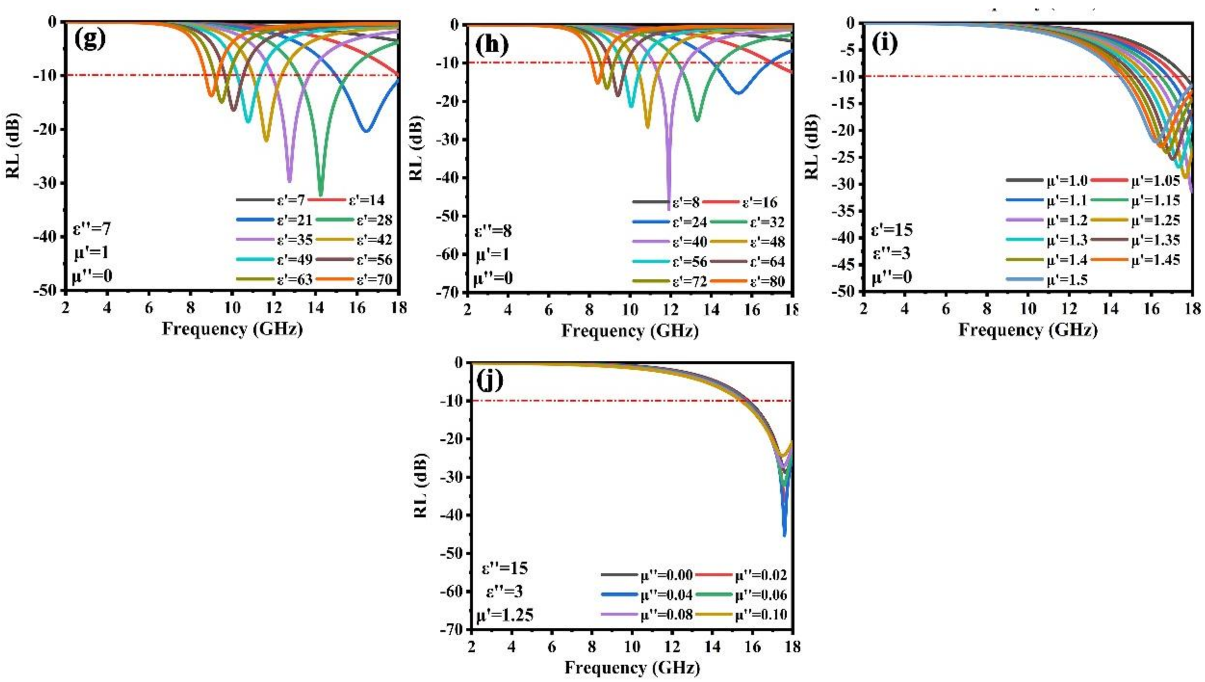

Fig. S6 Deductive calculation of reflection loss under 1.00 mm thickness

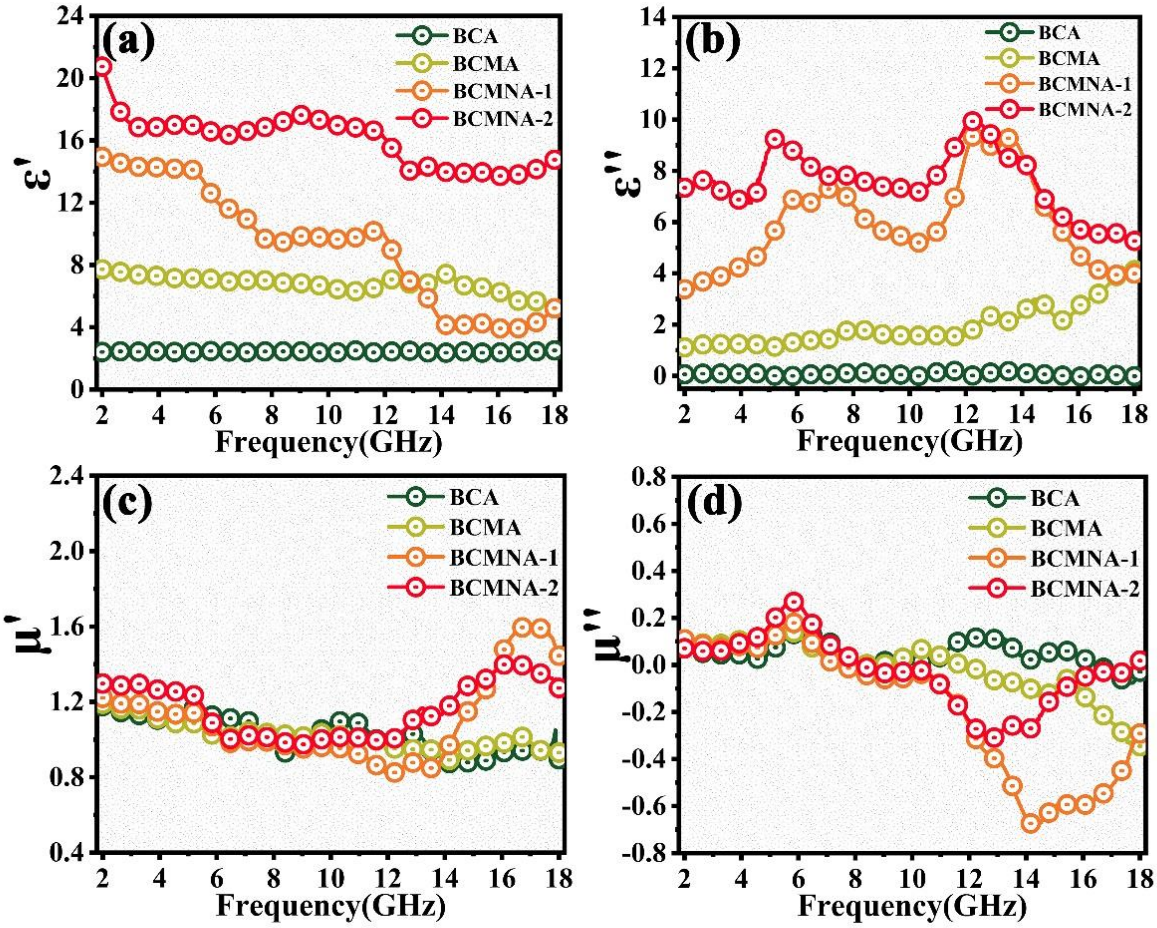

Fig. S7 The EMW parameters of samples, the real ε′ (**a**) and imaginary ε″ (**b**) parts of the complex permittivity; real μ′ (**c**) and imaginary μ″ (**d**) parts of the complex permeability

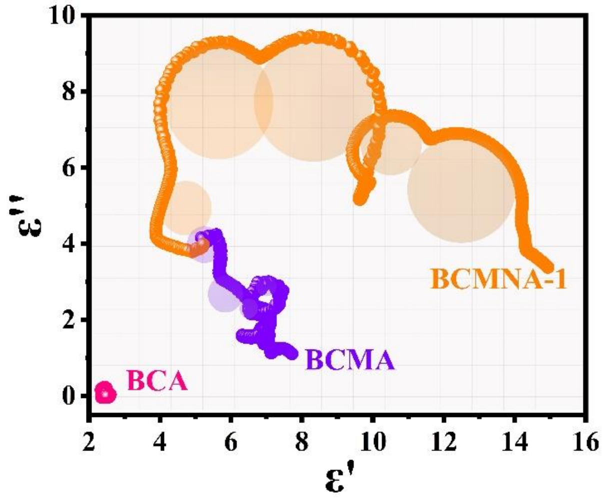

Fig. S8 Cole-Cole semicircle of BCA, BCMA and BCMNA-1

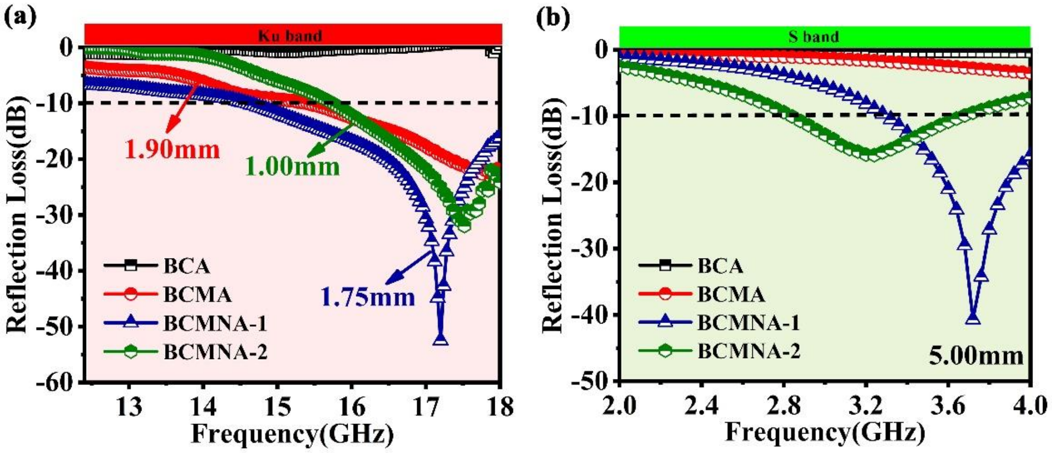

Fig. S9 2D RLs-f curves of samples at (**a**) Ku-band and (**b**) S-band

Table S1 EMW absorbing properties of similar materials

Sample	|RL| (dB)	Bandwidth (GHz)	Thickness (mm)	Refs.
MX/Gelatin AG	59.5	6.2	2.00	[S1]
TiO_2/MX/ rGO AG	65.3	2.8	2.50	[S2]
MX/rGO AG	49.1	2.9	1.20	[S3]
MX/polyimide AG	41.8	6.5	2.57	[S4]
Cellulose/MX AG	43.4	4.5	2.00	[S5]
MX/Fe_3O_4	57.3	2.0	1.90	[S6]
MX/$NiCo_2O_4$	50.9	1.5	2.18	[S7]
MX/Ni Chain/ZnO	35.1	3.0	2.80	[S8]
RGO/MX/Fe_3O_4	51.2	4.7	2.90	[S9]
MX/FeCo	43.7	0.9	2.90	[S10]
CoNi/RGO AG	53.3	3.5	2.95	[S11]
Ni/GO AG	47.0	6.8	2.00	[S12]
Ni-NiO/carbon AG	41.9	3.5	1.50	[S13]
Co/crystalline carbon/carbon AG	43.0	2.6	1.50	[S14]
Fe_3O_4/ZIF-67/wood AG	23.4	4.5	1.50	[S15]
BCMNA-2	31.9	2.3	1.00	**this work**

MX: MXene AG: aerogel RGO: graphene oxide

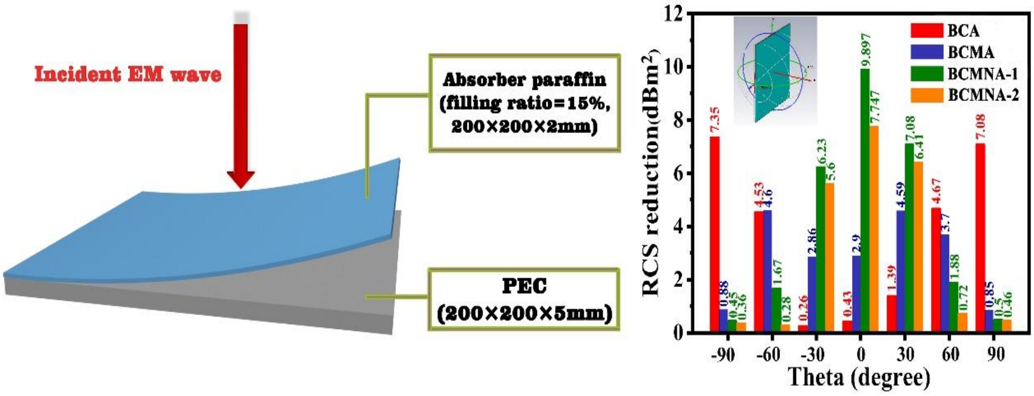

Fig. S10 RCS schematic diagram and RCS reduction achieved by subtracting the samples with the PEC

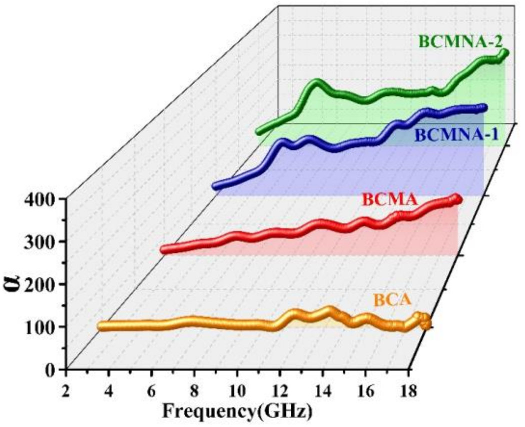

Fig. S11 Attenuation constant of samples

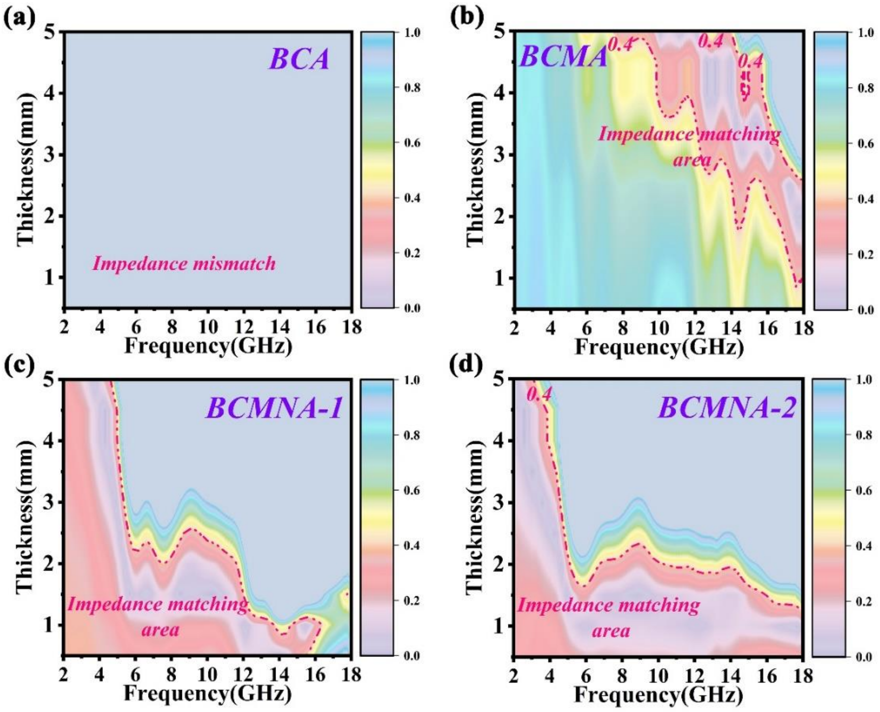

Fig. S12 Calculated delta value maps: (**a**) BCA, (**b**) BCMA, (**c**) BCMNA-1, and (**d**) BCMNA-2

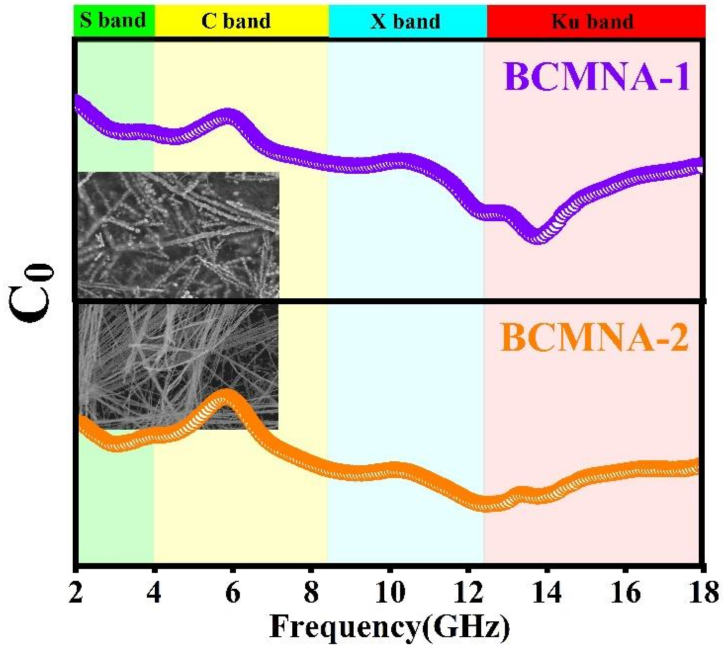

Fig. S13 C_0 value of BCMNA-1 and BCMNA-2

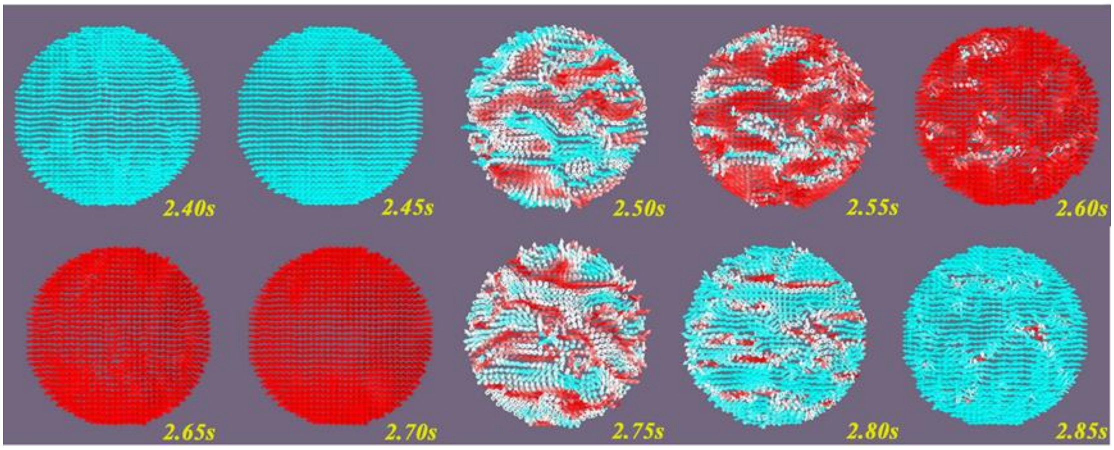

Fig. S14 Micromagnetic simulation of single Ni particular at 2 GHz

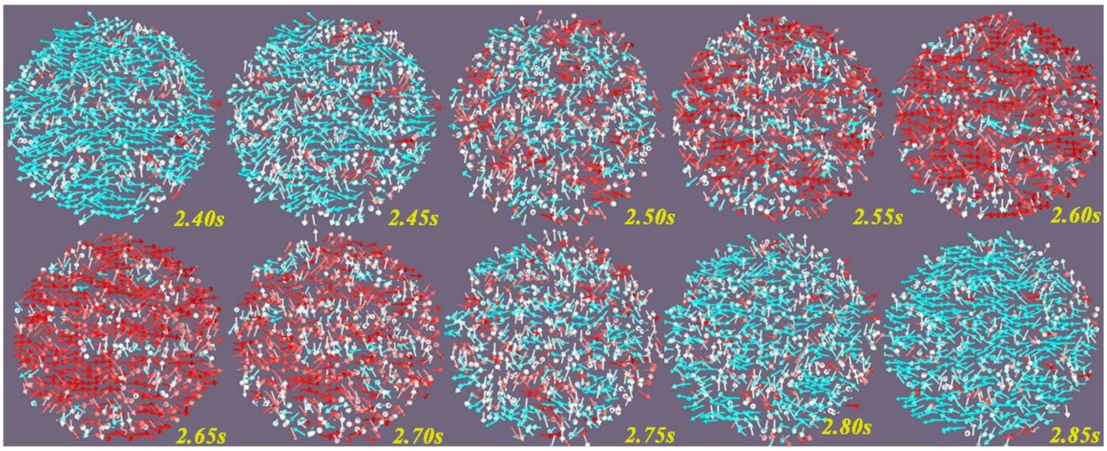

Fig. S15 Micromagnetic simulation of single Ni particular at 10 GHz

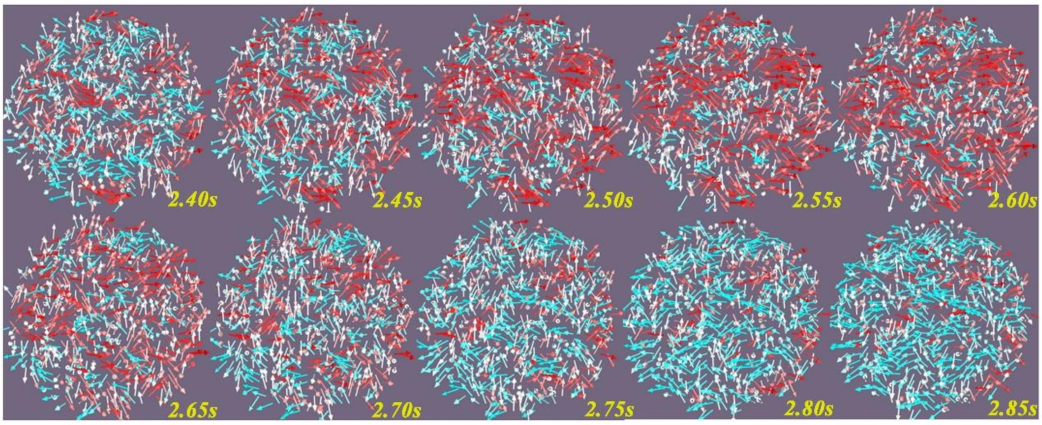

Fig. S16 Micromagnetic simulation of single Ni particular at 18 GHz

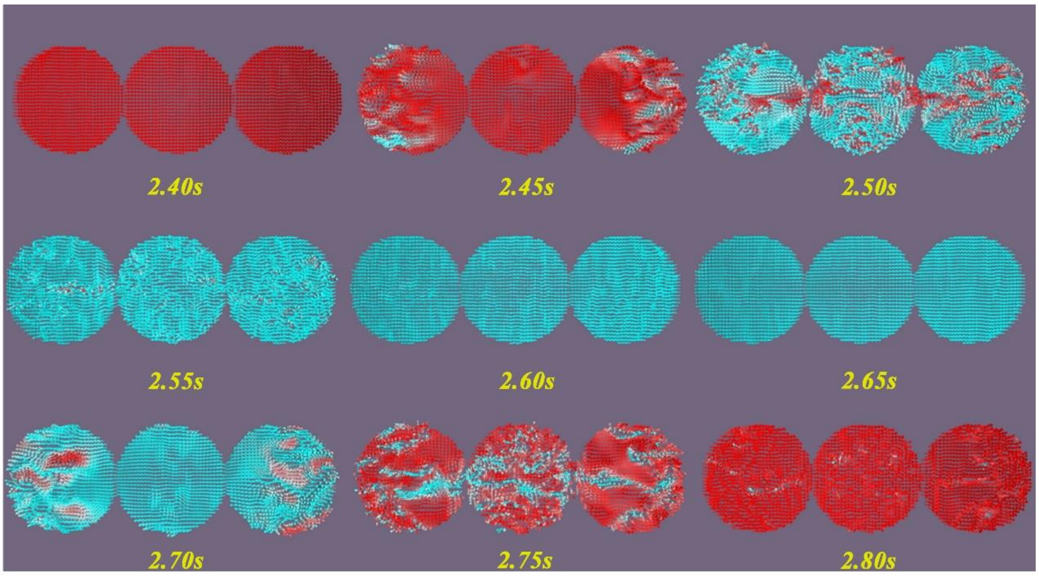

Fig. S17 Micromagnetic simulation of Ni chain at 2 GHz

Supplementary References

[S1] M. Yang, Y. Yuan, Y. Li, X. Sun, S. Wang et al., Anisotropic electromagnetic absorption of aligned $Ti_3C_2T_x$ MXene/gelatin nanocomposite aerogels. ACS Appl. Mater. Interfaces **12**(29), 33128-33138 (2020). https://doi.org/10.1021/acsami.0c09726

[S2] Y. Tong, M. He, Y.M. Zhou, S.X. Nie, X. Zhong et al., Three-dimensional hierarchical architecture of the $TiO_2/Ti_3C_2T_x$/RGO ternary composite aerogel for enhanced electromagnetic wave absorption. ACS Sustain. Chem. Eng. **6**(7), 8212-8222 (2018). https://doi.org/10.1021/acssuschemeng.7b04883

[S3] Y. Li, F.B. Meng, Y. Mei, H.G. Wang, Y.F. Guo et al., Electrospun generation of $Ti_3C_2T_x$ MXene@graphene oxide hybrid aerogel microspheres for tunable high-performance microwave absorption. Chem. Eng. J. **391**, 123512 (2020). https://doi.org/10.1016/j.cej.2019.123512

[S4] Y. Dai, X. Wu, Z. Liu, H.B. Zhang, Z.Z. Yu, Highly sensitive, robust and anisotropic MXene aerogels for efficient broadband microwave absorption. Compos. Part B Eng. **200**, 108263 (2020). https://doi.org/10.1016/j.compositesb.2020.108263

[S5] Y. Jiang, X. Xie, Y. Chen, Y.J. Liu, R. Yang et al., Hierarchically structured cellulose aerogels with interconnected MXene networks and their enhanced microwave

absorption properties. J. Mater. Chem. C **6**(32), 8679-8687 (2018). https://doi.org/10.1039/C8TC02900H

[S6] P.J. Liu, Z.J. Yao, V.M.H. Ng, J.T. Zhou, L.B. Kong et al., Facile synthesis of ultrasmall Fe_3O_4 nanoparticles on MXenes for high microwave absorption performance. Compos. Part A Appl. Sci. Manuf. **115**, 371-382 (2018). https://doi.org/10.1016/j.compositesa.2018.10.014

[S7] T.Q. Hou, B.B. Wang, M.L. Ma, A.L. Feng, Z.Y. Huang et al., Preparation of two-dimensional titanium carbide ($Ti_3C_2T_x$) and $NiCo_2O_4$ composites to achieve excellent microwave absorption properties. Compos. Part B Eng. **180**, 107577 (2020). https://doi.org/10.1016/j.compositesb.2019.107577

[S8] S. Wang, D. Li, Y. Zhou, L. Jiang, Hierarchical $Ti_3C_2T_x$ MXene/Ni chain/ZnO array hybrid nanostructures on cotton fabric for durable self-cleaning and enhanced microwave absorption. ACS Nano **14**(7), 8634-8645 (2020). https://doi.org/10.1021/acsnano.0c03013

[S9] Y. Cui, K. Yang, J. Wang, T. Shah, Q. Zhang et al., Preparation of pleated RGO/MXene/Fe_3O_4 microsphere and its absorption properties for electromagnetic wave. Carbon **172**, 1-14 (2021). https://doi.org/10.1016/j.carbon.2020.09.093

[S10] X. Li, C. Wen, L. Yang, R. Zhang, X. Li et al., MXene/FeCo films with distinct and tunable electromagnetic wave absorption by morphology control and magnetic anisotropy. Carbon **175**, 509-518 (2021). https://doi.org/10.1016/j.carbon.2020.11.089

[S11] Y. Zhao, X. Zuo, Y. Guo, H. Huang, H. Zhang et al., Structural engineering of hierarchical aerogels comprised of multi-dimensional gradient carbon nanoarchitectures for highly efficient microwave absorption. Nano-Micro Lett. **13**, 144 (2021). https://doi.org/10.1007/s40820-021-00667-7

[S12] F. Sun, Q. Liu, Y. Xu, X. Xin, Z. Wang et al., Attapulgite modulated thorny nickel nanowires/graphene aerogel with excellent electromagnetic wave absorption performance. Chem. Eng. J. **415**, 128976 (2021). https://doi.org/10.1016/j.cej.2021.128976

[S13] L. Wang, M. Liu, G. Wang, B. Dai, F. Yu et al., An ultralight nitrogen-doped carbon aerogel anchored by Ni-NiO nanoparticles for enhanced microwave adsorption performance. J. Alloys Comp. **776**, 43-51 (2019). https://doi.org/10.1016/j.jallcom.2018.10.214

[S14] H.B. Zhao, J.B. Cheng, Y.Z. Wang, Biomass-derived Co@crystalline carbon@carbon aerogel composite with enhanced thermal stability and strong microwave absorption performance. J. Alloys Comp. **736**, 71-79 (2018). https://doi.org/10.1016/j.jallcom.2017.11.120

[S15] L. Xu, Y. Xiong, B. Dang, Z. Ye, C. Jin et al., In-situ anchoring of Fe_3O_4/ZIF-67 dodecahedrons in highly compressible wood aerogel with excellent microwave absorption properties. Mater. Des. **182**, 108006 (2019). https://doi.org/10.1016/j.matdes.2019.108006
